# Associations between life-course household wealth mobility and adolescent physical growth, cognitive development and emotional and behavioral problems: A birth cohort in rural western China

**DOI:** 10.3389/fpubh.2023.1061251

**Published:** 2023-02-02

**Authors:** Jiaxin Tian, Yingze Zhu, Shuang Liu, Liang Wang, Qi Qi, Qiwei Deng, Amanuel Kidane Andegiorgish, Mohamed Elhoumed, Yue Cheng, Chi Shen, Lingxia Zeng, Zhonghai Zhu

**Affiliations:** ^1^Department of Epidemiology and Biostatistics, School of Public Health, Xi'an Jiaotong University Health Science Center, Xi'an, Shaanxi, China; ^2^Sichuan Center for Disease Control and Prevention, Institute of Tuberculosis Control and Prevention, Chengdu, China; ^3^Department of Nutrition and Food Safety Research, School of Public Health, Xi'an Jiaotong University Health Science Center, Xi'an, Shaanxi, China; ^4^School of Public Policy and Administration, Xi'an Jiaotong University, Shaanxi, China; ^5^Key Laboratory of Environment and Genes Related to Diseases, Xi'an Jiaotong University, Ministry of Education, Xi'an, Shaanxi, China

**Keywords:** birth cohort, household wealth mobility, adolescent, physical growth, cognition, behavioral health

## Abstract

**Background:**

Parental household wealth has been shown to be associated with offspring health conditions, while inconsistent associations were reported among generally healthy population especially in low- and middle- income countries (LMICs). Whether the household wealth upward mobility in LMICs would confer benefits to child health remains unknown.

**Methods:**

We conducted a prospective birth cohort of children born to mothers who participated in a randomized trial of antenatal micronutrient supplementation in rural western China. Household wealth were repeatedly assessed at pregnancy, mid-childhood and early adolescence using principal component analysis for household assets and dwelling characteristics. We used conditional gains and group-based trajectory modeling to assess the quantitative changes between two single-time points and relative mobility of household wealth over life-course, respectively. We performed generalized linear regressions to examine the associations of household wealth mobility indicators with adolescent height- (HAZ) and body mass index-for-age and sex z score (BAZ), scores of full-scale intelligent quotient (FSIQ) and emotional and behavioral problems.

**Results:**

A total of 1,188 adolescents were followed, among them 59.9% were male with a mean (SD) age of 11.7 (0.9) years old. Per SD conditional increase of household wealth z score from pregnancy to mid-childhood was associated with 0.11 (95% CI 0.04, 0.17) SD higher HAZ and 1.41 (95% CI 0.68, 2.13) points higher FSIQ at early adolescence. Adolescents from the household wealth Upward trajectory had a 0.25 (95% CI 0.03, 0.47) SD higher HAZ and 4.98 (95% CI 2.59, 7.38) points higher FSIQ than those in the Consistently low subgroup.

**Conclusion:**

Household wealth upward mobility particularly during early life has benefits on adolescent HAZ and cognitive development, which argues for government policies to implement social welfare programs to mitigate or reduce the consequences of early-life deprivations. Given the importance of household wealth in child health, it is recommended that socioeconomic circumstances should be routinely documented in the healthcare record in LMICs.

## 1. Introduction

The national economic growth in low- and middle- income countries (LMICs) has been rapidly developing for decades, potentially resulting in the improvement of child health. According to country-level data, the global under-five mortality rate substantially decreased by 53%, from 90.6 in 1990 to 42.5 deaths per 1,000 livebirths in 2015 (1). In general population, individual households have varying patterns and/or degrees of household wealth mobility. Based on demographic and health surveys in 39 LMICs, Winskill et al. (2) reported that children in the poorest households had a higher probability of co-occurring fever, acute respiratory infection, diarrhea and wasting. However, a study using data of 121 demographic and health surveys in 36 LMICs between 1990 and 2011 observed a quantitatively very small to null association between increases in per-head gross domestic product and reductions in early childhood malnutrition (3).

These discrepancies may be explained by the single-time point measurement of household wealth and cross-sectional nature of demographic and health surveys among these studies, which are unable to capture the mobility of individual household wealth. We only noted two studies conducted in high-income countries that measured socioeconomic status (SES) at multiple-time points, which, however, manually categorized the sample into subgroups by merging the low-, medium-, and high-SES at single-time point in an un-nuanced manner. They both reported that upward shift of household SES from baseline had benefits on later cardiovascular health (4, 5). However, household wealth as the largest contributor to child health among individual SES measures (6, 7), the associations between household wealth mobility and child heath remain unclear. Besides, household wealth can vary by years as compared to stable parental education and occupation. In addition, as the theory of Developmental Origins of Health and Disease describes that exposures to deprivations during early life may lay the foundations for long-term health (8), it remains unclear whether the upward mobility of postnatal household wealth would buffer against the negative effects of prenatal disadvantaged environment on child long-term health (4). Finally, household wealth and its mobility may exert positive or negative effects on child and adolescent specific health outcome (9).

In this study, we used data from a birth cohort in rural western China where national economy has been rapidly developing for decades. In our village setting, individual families have wider range and higher diversities of household wealth as compared to those in eastern metropolitan cities in last decade, i.e., during our study period, providing the unique opportunity to assess the household wealth mobility. We prospectively followed participants at birth, mid-childhood (7–9 y) and early adolescence (10–14 y) and repeatedly assessed household wealth at each visit. We aimed to examine the associations of household wealth at single-time point, conditional increase between two single-time points and life-course relative mobilities (trajectories) from pregnancy to early adolescence with adolescent multiple health outcomes, including physical growth, cognitive development, and emotional and behavioral problems.

## 2. Materials and methods

### 2.1. Study design and participant

We conducted a prospective birth cohort of children born to mothers who participated in a cluster-randomized, double-blind trial in rural western China conducted between August 2002 and February 2006 (ISRCTN08850194) (10). Briefly, all pregnant women from every village in two counties were eligible to enroll in this trial and were randomized to take a daily capsule of folic acid, iron/folic acid, or multiple micronutrients until delivery. Among 4,488 singleton live births eligible to enroll in long-term follow-up after excluding birth defects, and/or deaths (online Supplementary Figure 1), we followed 1,744 children at mid-childhood (age 7–10 years) between 2012 and 2013, and among them, 1,188 were followed at early adolescence (age 10–14 years) between June-December 2016. The procedure details of the parent trial and follow-up studies were described elsewhere (10–12). The trial and follow-up evaluation protocols were approved by the Ethics Committee in Xi'an Jiaotong University Health Science Center. Written informed consent was obtained from the biological parents or caregivers, and verbal consent was obtained from all the participants depending on their age.

### 2.2. Household wealth index

We repeatedly assessed household wealth at enrollment of parent trial (<28 gestational weeks), mid-childhood and early adolescence, which was derived from principal component analysis for household assets and dwelling characteristics. The following items were included at pregnancy: (i) goods, bicycle, motor, orchard, radio, TV/VCD, refrigerator, washer, poultry, goat/sheep/pig, cattle/cow and car/tractor; (ii) characteristics of the house, house types (soil cave-dwelling, brick-cave dwelling, soil wall, brick/concrete wall, and apartment), materials for the floor, and availability of electricity, running water and household toilet. In addition to these, phone, computer, air conditioner and automatic water heater were included at mid-childhood and early adolescence. Briefly, household assets and dwelling characteristics were classified into Having/Yes and Not having/No. The household wealth index was priorly constructed and locally validated (13). We categorized the household wealth index to indicate low-, medium- and high-wealth households by its tercile.

### 2.3. Measurements of adolescent health at adolescence-stage visit

#### 2.3.1. Physical growth

All anthropometric measures were taken by the same filed worker following standardized procedures in a local school classroom. Standing height was measured to 1 mm precision using a stadiometer (SZG-210, Shanghai JWFU Medical Apparatus Corporation) and weight after losing heavy clothes was measured to nearest the 0.1 kg (BC-420, Tanita Corporation, Tokyo, Japan). Height-for-age and sex *z* score (HAZ) and body mass index-for-age and sex *z* score (BAZ) were derived using World Health Organization growth standards (14). Adolescent stunting and overweight/obesity was defined as HAZ <−2 standard deviation (SD) and BAZ > +1 SD, respectively. Body mass index (kg/m^2^) was calculated as weight in kilograms divided by the square of height in meters.

#### 2.3.2. Cognitive development

We used a validated Chinese version of the Wechsler Intelligence Scale for Children, Fourth Edition (WISC-IV) (15). The full-scale intelligent quotient (FSIQ) was derived to represent adolescent general cognitive development. Besides, other aspects of adolescent cognitive development including verbal comprehension (VCI), perceptual reasoning (PRI), working memory (WMI), and processing speed index (PSI) were derived.

#### 2.3.3. Emotional and behavioral problems

Adolescents actively completed the scale of the Chinese version of Achenbach Youth's Self-Report (2001 version) under the guidance of field workers (16). Three continuous scores were derived, with lower scores indicating better emotional and behavioral outcome. Internalizing score was composed of withdrawn, anxious/depressed and somatic complaints, externalizing score was composed of delinquent/rule-breaking and aggressive behavior, and the total behavioral problem score was composed of all symptoms above and social problem, thought problem and attention problem. Both of the cognitive development and emotional and behavioral problem assessments were administrated in a local school meeting room free of distraction.

### 2.4. Other covariables

We collected the following covariables by face-to-face interview using standard procedures in the parent trial. We included parent age (continuous), education (<3 years, primary, secondary, ≥high school) and occupation (farmer, others), antenatal randomized regimens with durations (folic acid or folic acid plus iron <180 days, folic acid plus iron ≥180 days, multiple micronutrients <180 days, and multiple micronutrients ≥180 days) accounting for prior findings (12), maternal parity (0, ≥1), maternal mid-upper arm circumference at enrollment (<21.5 cm, ≥21.5 cm), and birth outcomes [small-for-gestational age by <10th centile by INTERGROWTH (17) and sex].

### 2.5. Statistical analyses

To assess the conditional increase of household wealth, we calculated the conditional gains of household wealth index between two single-time points, which was the standardized residual of regressing household wealth index at prior time point on household wealth index at later (18). To assess the life-course relative mobility of household wealth from pregnancy to early adolescence, we performed group-based trajectory modeling that assigned individuals with similar features of household wealth mobility trajectories into distinct, exclusive subgroups (19), using the “traj” command implemented in Stata software. Models with two or more subgroups were conducted after accounting for the varying trajectory shapes of linear, quadratic and/or cubic terms. Data-based parameters and principles were applied to decide the final subgroups (19), including (i) Bayesian and Akaike information criterion value, (ii) average of the posterior probabilities of group membership for individuals assigned to each group >0.7, (iii) odds of correct classification based on the posterior probabilities of group membership >5, and (iv) minimizing overlap in confidence intervals (CIs) and capturing the distinctive features of the data as parsimonious as possible.

We took adolescent HAZ, BAZ, FSIQ, and scores of total behavioral problems, externalizing and internalizing behavioral problems as primary outcomes, and other aspects of cognitive development and emotional and behavioral problems as secondary outcomes, respectively. We performed generalized linear regressions with Gaussian distribution and identity link to examine the associations of household wealth mobility indicators with adolescent health outcomes separately. The adjusted mean differences with their 95% CIs were estimated after including the covariables above. In addition, we performed stratified analyses by parental education (low and high educational level) and adolescent sex (male and female) after obtaining the interaction *P*-values that were estimated from likelihood ratio tests comparing models including and excluding the interaction terms.

For sensitive analyses addressing the lost to follow-up, we conducted inverse probability weighting, and randomly sampled the lowest and highest 80% wealth households at pregnancy and repeated the analyses for primary outcomes. The weight of each participant is given by the inverse of the predicted probability for followed participant in a logistic regression model, which included parental education, occupation and age, maternal parity and mid-upper arm circumference, randomized regimens by duration, small-for-gestational age, and adolescent sex and age. In addition, we used E-value approach to assess the impact extent of potential unmeasured confounder on affecting the estimates above (20). All statistical analyses were conducted in Stata 15.0 (Stata Corp, College Station, Texas, USA). A two-sided *P*-value < 0.05 was considered statistically significant.

## 3. Results

### 3.1. Background characteristics

Among 1,188 adolescents included in the final analyses (Table 1), 59.9% were male, and the mean age was 11.7 (SD, 0.9) years old. Majority of their parents had secondary education and lived on farming. The percentage of adolescent stunting and overweight/obesity was 2.3% (27/1,181) and 14.2% (166/1,167), respectively. The mean (SD) of adolescent HAZ, BAZ, FSIQ, total behavioral problem score, internalizing and externalizing score was 0.07 (1.07), −0.27 (1.15), 97.2 (12.4) points, 49.0 (24.1) points, 11.2 (7.6) points, and 8.4 (7.1) points, respectively. Adolescents born to parents who had higher education level, were non-farmers and came from high-wealth households were more likely to be lost to follow-up (Table 1). However, the characteristics of birth outcomes between adolescents followed and those lost to follow-up were balanced.

**Table 1:** Comparison of background characteristics between adolescents followed and those lost to follow-up in a birth cohort in rural western China.

**Factors**	**Adolescents followed from pregnancy through mid-childhood into early adolescence/number (%)**	**Adolescents lost to follow-up /number (%)**	***P*-values**
*n*	1,188 (100.0)	3,300 (100.0)	
Maternal age years/mean (SD)	24.8 (4.5)	24.6 (4.3)	0.18
**Maternal education**			<0.001
<3 years	77 (6.5)	173 (5.3)	
Primary	371 (31.3)	798 (24.3)	
Secondary	600 (50.6)	1,811 (55.1)	
High school and above	137 (11.6)	503 (15.3)	
Maternal occupation			<0.001
Farmer	1,030 (87.1)	2,710 (82.7)	
Others	152 (12.9)	565 (17.3)	
Paternal age (years)/Mean (SD)	28.0 (4.2)	27.8 (4.1)	0.10
**Paternal education**			<0.001
<3 years	20 (1.7)	40 (1.2)	
Primary	188 (15.9)	387 (11.8)	
Secondary	735 (62.0)	2,033 (61.9)	
High school and above	243 (20.5)	824 (25.1)	
**Paternal occupation**			<0.001
Farmer	951 (80.3)	2,400 (72.9)	
Others	234 (19.7)	890 (27.1)	
Household wealth index at pregnancy/enrollment^a^	−0.14 (1.4)	0.09 (1.5)	<0.001
Low (Q1)	412 (34.7)	1,021 (30.9)	0.003
Medium (Q2)	429 (36.1)	1140 (34.6)	
High (Q3)	347 (29.2)	1,139 (34.5)	
**Parity at enrollment**			0.19
0	757 (63.7)	2,172 (65.8)	
≥1	431 (36.3)	1,128 (34.2)	
Maternal MUAC (cm)			0.341
<21.5	225 (19.1)	582 (17.9)	
≥21.5	953 (80.9)	2,678 (82.1)	
**Randomized regimens**			0.12
Folic acid	732 (34.6)	853 (36.0)	
Folic acid plus iron	676 (32.0)	795 (33.5)	
Multiple micronutrient	707 (33.4)	725 (30.5)	
**Offspring sex**			<0.001
Male	712 (59.9)	1,773 (53.7)	
Female	476 (40.1)	1,527 (46.3)	
Birth weight (gram) /Mean (SD)	3,205 (416)	3,194 (416)	0.46
Gestational weeks at delivery /Mean (SD)	39.8 (1.6)	39.8 (1.7)	0.63
Preterm (<37 gestational weeks)	50 (4.2)	158 (4.8)	0.42
Low birth weight (<2,500 g)	47 (4.1)	99 (3.2)	0.14
Small-for-gestational age (<10th)	138 (12.4)	374 (12.4)	0.99
Age at adolescence (years)/mean (SD)	11.7 (0.9)	11.7 (0.9)	0.55

SD, standard deviation; MUAC, mid-upper arm circumference.

^a^Household wealth at enrollment was derived from principal component analysis for household assets and dwelling characteristics, and was further categorized by its terciles.

### 3.2. Household wealth mobility and adolescent health

#### 3.2.1. Household wealth at single-time point

As shown in Table 2, per SD increase of household wealth index at pregnancy was associated with 0.09 (95% CI 0.04, 0.14) SD higher HAZ, 0.71 (95% CI 0.11, 1.31) points higher FSIQ, and −1.54 (95% CI −2.84, −0.24) points lower scores of total behavioral problems and −0.47 (95% CI −0.85, −0.09) points lower scores of externalizing behavioral problems at early adolescence. Similar results were observed for household wealth index at mid-childhood and early adolescence, and for other aspects of adolescent cognitive development (Supplementary Table 1). While, we observed null associations of household wealth at mid-childhood and early adolescence with adolescent socioemotional scores, respectively (Table 2 and Supplementary Table 2).

**Table 2:** Associations between household wealth index at single-time point and adolescent HAZ, BAZ, cognitive development, and emotional and behavioral problems in a birth cohort in rural western China (*n* = 1,188).

**Household wealth index**	***Z*** **scores of physical growth**^**a**^		**Cognitive development^a^**	**Scores of emotional and behavioral problems** ^**a**^		
	**HAZ**	**BAZ**	**FSIQ**	**Total problem**	**Internalizing**	**Externalizing**
Pregnancy/per SD	0.09 (0.04, 0.14)	0.03 (−0.03, 0.09)	0.71 (0.11, 1.31)	−1.54 (−2.84, −0.24)	−0.31 (−0.73, 0.10)	−0.47 (−0.85, −0.09)
Low (Q1)	Ref.	Ref.	Ref.	Ref.	Ref.	Ref.
Medium (Q2)	0.06 (−0.09, 0.22)	−0.02 (−0.19, 0.15)	0.01 (−1.67, 1.68)	−3.09 (−6.69, 0.51)	−0.46 (−1.62, 0.69)	−0.60 (−1.65, 0.45)
High (Q3)	0.24 (0.06, 0.42)	0.12 (−0.08, 0.32)	2.43 (0.41, 4.45)	−5.24 (−9.62, −0.87)	−1.30 (−2.70, 0.10)	−1.75 (−3.02, −0.47)
Mid–childhood/per SD	0.10 (0.06, 0.15)	0.03 (−0.02, 0.08)	1.18 (0.70, 1.67)	−0.002 (−1.07, 1.07)	−0.11 (−0.45, 0.23)	−0.06 (−0.37, 0.25)
Low (Q1)	Ref.	Ref.	Ref.	Ref.	Ref.	Ref.
Medium (Q2)	0.16 (0.01, 0.31)	0.10 (−0.07, 0.27)	0.14 (−1.54, 1.81)	0.46 (−3.19, 4.12)	0.08 (−1.09, 1.24)	0.12 (−0.94, 1.19)
High (Q3)	0.38 (0.22, 0.55)	0.07 (−0.12, 0.25)	3.37 (1.53, 5.21)	−0.32 (−4.36, 3.72)	−0.50 (−1.79, 0.80)	−0.23 (−1.41, 0.95)
Early adolescence/per SD	0.11 (0.06, 0.16)	0.05 (0.003, 0.11)	1.48 (0.97, 1.99)	−0.48 (−1.60, 0.64)	−0.28 (−0.64, 0.08)	−0.14 (−0.47, 0.19)
Low (Q1)	Ref.	Ref.	Ref.	Ref.	Ref.	Ref.
Medium (Q2)	0.22 (0.07, 0.37)	0.24 (0.08, 0.41)	1.14 (−0.48, 2.75)	−2.12 (−5.66, 1.43)	−0.99 (−2.13, 0.14)	−0.04 (−1.08, 1.00)
High (Q3)	0.34 (0.17, 0.51)	0.14 (−0.05, 0.33)	5.16 (3.27, 7.05)	−1.68 (−5.84, 2.48)	−1.12 (−2.46, 0.21)	−0.48 (−1.69, 0.74)

HAZ, height-for- age and sex z score; BAZ, body mass index-for- age and sex z score; FSIQ, full-scale intelligent quotient.

^a^Data are presented with adjusted mean differences and their 95% confidence intervals. The adjustments included parental education, occupation and age, maternal parity and mid-upper arm circumference, randomized regimens by duration, small-for-gestational age, and adolescent sex and age.

#### 3.2.2. Conditional increase of household wealth between two single-time points

We observed positive associations of conditional increase/gain of household wealth between two single-time points, i.e., quantitative increase of household wealth, with adolescent HAZ and cognitive development, while null associations for BAZ and scores of emotional and behavioral problems in Table 3 and Supplementary Tables 1, 2. Specifically, per SD of household wealth conditional increase from pregnancy to mid-childhood was associated with 0.11 (95% CI 0.04, 0.17) SD higher HAZ and 1.41 (95% CI 0.68, 2.13) points higher FSIQ at early adolescence, respectively. Similar associations between conditional increase of household wealth from mid-childhood to early adolescence and adolescent HAZ and cognitive development (FSIQ and other aspects) were observed.

**Table 3:** Associations between conditional gains of household wealth among periods and adolescent HAZ, BAZ, cognitive development and emotional and behavioral problems in a birth cohort in rural western China (*n* = 1,188).

**Household wealth**	***Z*** **scores of physical growth**^**a**^		**Cognitive development^a^**	**Scores of emotional and behavioral problems** ^**a**^		
	**HAZ**	**BAZ**	**FSIQ**	**Total problem**	**Internalizing**	**Externalizing**
Gains from pregnancy to mid–childhood/per SD	0.11 (0.04, 0.17)	0.03 (−0.05, 0.10)	1.41 (0.68, 2.13)	0.69 (−0.90, 2.28)	−0.02 (−0.53, 0.49)	0.12 (−0.34, 0.59)
Low (Q1)	Ref.	Ref.	Ref.	Ref.	Ref.	Ref.
Medium (Q2)	0.04 (−0.11, 0.19)	0.07 (−0.10, 0.23)	0.87 (−0.79, 2.53)	0.47 (−3.18, 4.12)	−0.17 (−1.33, 1.00)	−0.10 (−1.17, 0.96)
High (Q3)	0.21 (0.05, 0.37)	0.06 (−0.12, 0.24)	2.82 (1.08, 4.57)	0.98 (−2.83, 4.79)	−0.23 (−1.45, 0.99)	0.24 (−0.87, 1.36)
<=0	Ref.	Ref.	Ref.	Ref.	Ref.	Ref.
>0	0.24 (0.11, 0.37)	0.05 (−0.09, 0.19)	1.98 (0.55, 3.41)	1.6 (−1.53, 4.73)	0.19 (−0.82, 1.19)	0.45 (−0.47, 1.36)
Gains from mid–childhood to early adolescence/per SD	0.06 (−0.01, 0.12)	0.05 (−0.02, 0.12)	1.07 (0.38, 1.76)	−0.43 (−1.93, 1.07)	−0.26 (−0.74, 0.22)	−0.08 (−0.52, 0.36)
Low (Q1)	Ref.	Ref.	Ref.	Ref.	Ref.	Ref.
Medium (Q2)	0.17 (0.02, 0.32)	0.28 (0.12, 0.45)	0.64 (−1.03, 2.30)	0.35 (−3.27, 3.97)	−0.02 (−1.18, 1.14)	0.11 (−0.95, 1.17)
High (Q3)	0.19 (0.04, 0.34)	0.19 (0.01, 0.36)	2.58 (0.87, 4.28)	0.01 (−3.71, 3.73)	−0.46 (−1.65, 0.73)	0.29 (−0.79, 1.38)
<=0	Ref.	Ref.	Ref.	Ref.	Ref.	Ref.
>0	0.11 (−0.02, 0.23)	0.12 (−0.02, 0.26)	1.69 (0.30, 3.07)	−0.53 (−3.56, 2.51)	−0.23 (−1.20, 0.74)	−0.17 (−1.06, 0.71)
Gains from pregnancy to early adolescence/per SD	0.11 (0.04, 0.18)	0.06 (−0.01, 0.14)	1.74 (1.00, 2.47)	0.06 (−1.56, 1.67)	−0.24 (−0.76, 0.28)	0.03 (−0.44, 0.50)
Low (Q1)	Ref.	Ref.	Ref.	Ref.	Ref.	Ref.
Medium (Q2)	0.14 (−0.005, 0.29)	0.23 (0.07, 0.39)	0.58 (−1.04, 2.19)	−0.99 (−4.54, 2.55)	−0.57 (−1.70, 0.57)	0.24 (−0.79, 1.28)
High (Q3)	0.29 (0.13, 0.45)	0.17 (−0.01, 0.35)	3.92 (2.14, 5.70)	−0.76 (−4.66, 3.13)	−0.91 (−2.16, 0.34)	−0.27 (−1.41, 0.87)
<=0	Ref.	Ref.	Ref.	Ref.	Ref.	Ref.
>0	0.15 (0.03, 0.28)	0.12 (−0.02, 0.26)	1.95 (0.55, 3.36)	−1.25 (−4.34, 1.83)	−0.76 (−1.75, 0.23)	−0.48 (−1.38, 0.42)

HAZ, height-for- age and sex z score; BAZ, body mass index-for- age and sex *z* score; FSIQ, full-scale intelligent quotient.

^a^Data are presented with adjusted mean differences and their 95% confidence intervals. The adjustments included parental education, occupation and age, maternal parity and mid-upper arm circumference, randomized regimens by duration, small-for-gestational age, and adolescent sex and age.

#### 3.2.3. Life-course trajectories (relative-scale mobility) of household wealth and adolescent health

We identified four distinct life-course trajectories of household wealth, which could be characterized as: (i) Consistently low (53.8% of the sample), (ii) Upward (11.4%), (iii) Downward (22.2%), and (iv) Consistently high (12.6%) (Supplementary Figure 2). The parameters of deciding the final trajectories were summarized in Supplementary Table 4 while accounting for the distinctive features of the data as parsimonious as possible.

Adolescents from the Upward subgroup had a 0.25 (95% CI 0.03, 0.47) SD higher HAZ than those in the Consistently low subgroup, while adolescents from the Downward subgroup had a −0.31 (95% CI −0.55, −0.07) SD lower HAZ as compared to those in the Consistently high subgroup (Table 4). The corresponding estimates were 4.98 (95% CI 2.59, 7.38) and −4.03 (−6.66, −1.41) points for adolescent FSIQ. Similar positive associations were observed for other aspects of adolescent cognitive development (Supplementary Table 1), while null associations for adolescent BAZ and scores of emotional and behavioral problems (Table 4 and Supplementary Table 2).

**Table 4:** Associations between life-course household wealth trajectories and adolescent HAZ, BAZ, cognitive development, and emotional and behavioral problems in a birth cohort in rural western China (*n* = 1,188).

**Household wealth**	***Z*** **scores of physical growth**^**a**^		**Cognitive development^a^**	**Scores of emotional and behavioral problems** ^**a**^		
	**HAZ**	**BAZ**	**FSIQ**	**Total problem**	**Internalizing**	**Externalizing**
Upward vs. Consistently low	0.25 (0.03, 0.47)	−0.01 (−0.25, 0.24)	4.98 (2.59, 7.38)	0.18 (−5.15, 5.52)	−0.56 (−2.27, 1.15)	0.08 (−1.47, 1.64)
Downward vs. Consistently low	0.19 (0.02, 0.36)	−0.03 (−0.23, 0.16)	2.62 (0.74, 4.51)	−1.47 (−5.67, 2.73)	−0.75 (−2.09, 0.60)	−0.47 (−1.70, 0.76)
Consistently high vs. Consistently low	0.49 (0.25, 0.73)	0.10 (−0.17, 0.37)	6.66 (4.00, 9.32)	−3.67 (−9.64, 2.30)	−1.07 (−2.98, 0.84)	−1.52 (−3.26, 0.22)
Upward vs. Consistently high	−0.24 (−0.52, 0.04)	−0.11 (−0.42, 0.21)	−1.67 (−4.76, 1.41)	3.86 (−3.17, 10.88)	0.51 (−1.74, 2.76)	1.60 (−0.45, 3.65)
Downward vs. Consistently high	−0.31 (−0.55, −0.07)	−0.13 (−0.40, 0.13)	−4.03 (−6.66, −1.41)	2.20 (−3.80, 8.20)	0.32 (−1.60, 2.24)	1.05 (−0.70, 2.80)
Upward vs. Downward	0.08 (−0.16, 0.32)	0.01 (−0.27, 0.30)	2.28 (−0.34, 4.90)	1.05 (−4.93, 7.03)	0.04 (−1.93, 2.00)	0.40 (−1.34, 2.14)

HAZ, height-for- age and sex z score; BAZ, body mass index-for- age and sex z score; FSIQ, full-scale intelligent quotient.

^a^Data are presented with adjusted mean differences and their 95% confidence intervals. The adjustments included parental education, occupation and age, maternal parity and mid-upper arm circumference, randomized regimens by duration, small-for-gestational age, and adolescent sex and age.

#### 3.2.4. Stratified analyses by parental education and adolescent sex

The *P*-values of interactions between parental education and adolescent sex and household wealth mobility indicators were presented in Supplementary Table 4, most of which were beyond 0.05. Further, we performed stratified analysis by maternal education (Supplementary Tables 5, 6), paternal education (Supplementary Tables 7, 8), and adolescent sex (Supplementary Tables 9, 10). The benefits of household wealth increase on adolescent HAZ and cognitive development were more pronounced among adolescents from households with higher maternal and paternal education. In addition, the benefits were doubled among adolescent females as compared with males.

### 3.3. Sensitivity analyses

Finally, the sensitivity analyses accounting for the lost to follow-up showed comparable results (Supplementary Tables 11–13) to those in Tables 2–4. The results of E-value approach indicated that an unmeasured confounder with strong strength would be required to explain away the observed associations (Supplementary Table 14), suggesting the robustness of our results.

## 4. Discussions

We observed that household wealth upward mobility particularly during early life was associated with adolescent higher HAZ and better cognitive development in a birth cohort in an undeveloped setting. Further, adolescents from wealthier households at pregnancy had lower (better) scores of emotional and behavioral problems. Although the interaction *P*-values did not reach statistical significance, the benefits of household wealth increase on adolescent HAZ and cognitive development were more pronounced among households with higher maternal or paternal education level. Furthermore, the corresponding benefits were doubled among female adolescents as compared with their counterparts.

We used household assets and dwelling characteristics to construct household wealth index, which is a robust measure in LMICs as compared to income and consumption, suffering from information bias. Our study is one of the few studies to comprehensively assess the mobility and trajectories of household wealth from pregnancy to early adolescence, evaluate adolescent health in multiple domains and examined their relationships in a nuanced manner. Our results show that postnatal household wealth conditional increase could confer the benefits to adolescent HAZ and cognitive development after accounting for the influence of household wealth at pregnancy. Besides, the effect size of household wealth conditional increase between pregnancy and mid-childhood seems to be larger than that between mid-childhood and early adolescence, although with confidence interval overlapping. This finding is in line with the hypothesis that the plasticity of child development is larger during early life, particularly during the first 1,000 days. Another explanation was that school education may have relatively larger impact on adolescent cognitive development as compared to household wealth mobility after mid-childhood/primary school age, given the national strategy of 9-year compulsory education well-implemented in China. In the meantime, some nutritional intervention programs were conducted among school, e.g., the Yingyangbao strategy widely covered in rural areas in China (21), which potentially buffer against the health consequences of disadvantaged families. In addition, on the relative scale (position change) of household wealth in our sample from pregnancy to early adolescence, our trajectory results show the consistency of wealth-driven health disparities in LMICs (22). Of note, adolescents from Upward household wealth trajectory had comparable HAZ and cognitive development to those from Consistently high household wealth trajectory. These results suggest that postnatal household wealth upward mobility may have long-lasting benefits on adolescent health, particularly for household wealth increase during early life. Majority of studies considered that the household wealth was a proxy of offspring access to health care, optimal diets, clean water and sanitation, home environment and other resources (23). Nevertheless, mediation analyses reported that the mediators above could not completely explain the associations between household wealth and health outcomes at later life (24–26). Among adults, Zhang and colleagues reported that healthy lifestyle only mediated a small proportion (3.0% to 12.3%) of the association between low SES and higher risk of mortality and cardiovascular diseases (27). These results suggest that poor household wealth at early life may have a direct or causal link to suboptimal life-course health outcomes, which may result from persistent structural changes due to deprivations during pregnancy (28). Our results that household wealth at pregnancy was associated with adolescent HAZ, cognitive development and emotional and behavioral health agree well with this hypothesis. Taken together, to improve child health and development in public health practices, much efforts should be made as early as practicable to lay the foundation and target the high-risk population in disadvantaged households. In public health implications, we suggested that deprived families should be included in social welfare programs at the beginning of pregnancy.

In addition, the potential benefits of household wealth increase over time may differ by specific health outcome. We observed consistent significance for adolescent HAZ and cognitive development which may result from the appropriate infant feeding, diets, stimulation, and home environment among higher wealth households, all of which have been shown to be causes of child linear growth and development (29). Prior study reported lower (better) scores of emotional and behavioral problems among children from wealthier households (30). Our study further contributes to the literature that higher household wealth at pregnancy but not postnatal household wealth increase has benefits on adolescent socioemotional outcomes. In addition, the transition of overweight/obesity from the wealthy to the poor along with the national economy increasing was documented in other LMICs (31). We only observed statistically positive associations of adolescent BAZ with household wealth at early adolescence, suggesting the likely minimal impact of household wealth mobility at early life on adolescent weight. However, we could not provide more details on the underlying mechanisms linking household wealth to a specific outcome, and mediation analyses examining corresponding mediators are needed in future. Overall, postnatal household wealth increase has some long-term benefits on adolescent health particularly for adolescents being born and raised in consistently wealthy families, which argue for government policies to implement social welfare programs such as cash transfer and health insurance to mitigate or reduce the consequences of early-life deprivations (32).

Furthermore, we examined the modifications of parent education and offspring sex on the relationship between household wealth mobilities and adolescent health, although majority of these interaction *P*-values were not statistically significant. In the present study, the benefits of postnatal household wealth increase on adolescent health were more pronounced among adolescents from households with higher parental education. Similarly, prior study reported that higher maternal education might buffer against the negative effects of higher household wealth on child overweight/obesity (33). Besides, Conger and colleagues reported that higher parent education level would increase their family investments on child care and consequently lead to the improvement of child health (34). We hypothesized that households with higher parental education were more likely to take advantage of the wealth and transfer resources into appropriate practices of child care, consequently improving adolescent health (35). As for the sex modification, the benefits of household wealth increase on adolescent health were observed both among female and male adolescents, but the effect sizes particularly for cognitive development were doubled among adolescent females. Prior study reported that adolescent females relate to males were more likely to follow the parenting on healthy life styles (36). Similarly, we previously reported a statistically significant interactions of maternal education and sex for adolescent anemia (37). In addition, the relationship of household wealth at pregnancy and adolescent emotional and behavioral health was only observed among males in the present study, which may be due to the statistical power, and/or that adolescent males had higher prevalence of externalizing behavioral problems as compared to females (38). However, the sex modification of the relationship between SES and children emotional and behavioral outcomes were not consistent in the literature (39), warranting confirmations in future studies.

Our findings have a few limitations. Firstly, adolescents were born to mothers who had participated in an antenatal micronutrient supplementation trial which might limit the generalization. However, this community-based trial enrolled all eligible pregnant women in every village and we adjusted for randomized regimens in all analyses. Besides, this micronutrient intervention strategy would be expected to weaken the tie between household wealth and adolescent health for its ability to reduce the wealth-driven equity (40). Secondly, as with other cohorts with long-term follow-up periods, loss of participants may lead to the selection bias. Households with higher parental education and wealth are more likely to move out of study area into cities and thus be lost to follow, resulting in the underestimates in our study. We have partly addressed this by performing inverse probability weighting and repeating analyses among the lowest and highest 80% wealth households at baseline, all of which showed comparable results. Thirdly, the household wealth index derived from assets and dwelling characteristics might be country-specific, although it was commonly used in LMICs. Besides, the wealth mobility defined by household wealth index may not indicate the actual increase of inflation-adjusted family income, which however mainly suggested wealth positional change among our participants. Finally, residual confounding was always possible due to the nature of observational design, and causal inference could be pursued under the counterfactual outcome framework in future studies as the reviewer suggested. However, our sensitivity analyses of E-value approach suggested that the contribution of unmeasured confounding to biasing our results was likely minimal.

Higher prenatal household wealth and postnatal household wealth increase particularly during early life had wide benefits on adolescent HAZ and cognitive development, and possibly socioemotional outcomes. To improve adolescent health and human capital outcomes, public health programs targeting at all life-course stages are warranted and should be accompanied by strategies to reach the most vulnerable populations at the beginning of pregnancy. Given the importance of household wealth and other related SES indicators in child health, it is recommended that socioeconomic circumstances should be routinely documented in the healthcare record.

## Data availability statement

The original contributions presented in the study are included in the article/Supplementary material, further inquiries can be directed to the corresponding authors.

## Ethics statement

The studies involving human participants were reviewed and approved by Ethics Committee in Xi'an Jiaotong University Health Science Center. Written informed consent to participate in this study was provided by the participants' legal guardian/next of kin.

## Author contributions

YC, LZ, and ZZ designed the study. JT, SL, LW, QQ, QD, AA, ME, and ZZ conducted the study. JT, YZ, and ZZ analyzed data and interpreted results. JT, YZ, and ZZ wrote the paper. LZ and ZZ had primary responsibility for final content. All authors reviewed, revised, and approved the final paper.

## References

[1] You D Hug L Ejdemyr S Idele P Hogan D Mathers C. Global, regional, and national levels and trends in under-5 mortality between 1990 and 2015, with scenario-based projections to 2030: a systematic analysis by the UN Inter-agency Group for Child Mortality Estimation. Lancet. (2015) 386:2275–86. 10.1016/S0140-6736(15)00120-8 26361942

[2] Winskill P Hogan AB Thwing J Mwandigha L Walker PGT Lambert B. Health inequities and clustering of fever, acute respiratory infection, diarrhoea and wasting in children under five in low- and middle-income countries: a demographic and health surveys analysis. BMC Med. (2021) 19:114. 10.1186/s12916-021-02018-0 34162389 PMC8223394

[3] Vollmer S Harttgen K Subramanyam MA Finlay J Klasen S Subramanian SV. Association between economic growth and early childhood undernutrition: evidence from 121 demographic and health surveys from 36 low-income and middle-income countries. Lancet Glob Health. (2014) 2:e225–34. 10.1016/S2214-109X(14)70025-7 25103063

[4] Poulton R Caspi A Milne BJ Thomson WM Taylor A Sears MR. Association between children's experience of socioeconomic disadvantage and adult health: a life-course study. Lancet. (2002) 360:1640–5. 10.1016/S0140-6736(02)11602-3 12457787 PMC3752775

[5] Sung J Song YM Hong KP. Relationship between the shift of socioeconomic status and cardiovascular mortality. Eur J Prev Cardiol. (2020) 27:749–57. 10.1177/2047487319856125 31180761

[6] Kumar R Paswan B. Changes in socio-economic inequality in nutritional status among children in EAG states, India. Public Health Nutr. (2021) 24:1304–17. 10.1017/S1368980021000343 33500017 PMC10195466

[7] Fagbamigbe AF Adebola OG Dukhi N Fagbamigbe OS Uthman OA. Exploring the socio-economic determinants of educational inequalities in diarrhoea among under-five children in low- and middle-income countries: a Fairlie decomposition analysis. Arch Public Health. (2021) 79:114. 10.1186/s13690-021-00639-8 34167581 PMC8223382

[8] Halfon N Larson K Lu M Tullis E Russ S. Lifecourse health development: past, present, and future. Matern Child Health J. (2014) 18:344–65. 10.1007/s10995-013-1346-2 23975451 PMC3890560

[9] Pathirana TI Jackson CA. Socioeconomic status and multimorbidity: a systematic review and meta-analysis. Aust N Z J Public Health. (2018) 42:186–94. 10.1111/1753-6405.12762 29442409

[10] Zeng L Dibley MJ Cheng Y Dang S Chang S Kong L. Impact of micronutrient supplementation during pregnancy on birth weight, duration of gestation, and perinatal mortality in rural western China: double blind cluster randomized controlled trial. BMJ. (2008) 337:a2001. 10.1136/bmj.a2001 18996930 PMC2577799

[11] Li C Zeng L Wang D Yang W Dang S Zhou J. Prenatal micronutrient supplementation is not associated with intellectual development of young school-aged children. J Nutr. (2015) 145:1844–9. 10.3945/jn.114.207795 26084366

[12] Zhu Z Cheng Y Zeng L Elhoumed M He G Li W. Association of antenatal micronutrient supplementation with adolescent intellectual development in rural western China: 14-year follow-up from a randomized clinical trial. JAMA Pediatr. (2018) 172:832–41. 10.1001/jamapediatrics.2018.1401 29987336 PMC6143069

[13] Zeng L Yan H Chen Z. Measurement of the living standards of family in rural area and relationship between wealth index and perinatal care status. Wei Sheng Yan Jiu. (2008) 6:714–7. Available online at: https://pubmed.ncbi.nlm.nih.gov/19239009/19239009

[14] Butte NF Garza C de Onis M. Evaluation of the feasibility of international growth standards for school-aged children and adolescents. J Nutr. (2007) 137:153–7. 10.1093/jn/137.1.153 17182818

[15] Chen H Keith TZ Weiss L Zhu J Li Y. Testing for multigroup invariance of second-order WISC-IV structure across China, Hong Kong, Macau, and Taiwan. Personal Individ Differ. (2010) 49:677–82. 10.1016/j.paid.2010.06.004

[16] Leung PW Kwong SL Tang CP Ho TP Hung SF Lee CC. Test-retest reliability and criterion validity of the Chinese version of CBCL, TRF, and YSR. J Child Psychol Psychiatry. (2006) 47:970–3. 10.1111/j.1469-7610.2005.01570.x 16930392

[17] Villar J Cheikh IL Victora CG Ohuma EO Bertino E Altman DG. International standards for newborn weight, length, and head circumference by gestational age and sex: the newborn cross-sectional study of the INTERGROWTH-21st Project. Lancet. (2014) 384:857–68. 10.1016/S0140-6736(14)60932-6 25209487

[18] Zhang X Tilling K Martin RM Oken E Naimi AI Aris IM. Analysis of “sensitive” periods of fetal and child growth. Int J Epidemiol. (2019) 48:116–23. 10.1093/ije/dyy045 29618044 PMC6380295

[19] Nagin DS Jones BL Passos VL Tremblay RE. Group-based multi-trajectory modeling. Stat Methods Med Res. (2018) 27:2015–23. 10.1177/0962280216673085 29846144

[20] VanderWeele TJ Ding P. Sensitivity analysis in observational research: introducing the E-value. Ann Intern Med. (2017) 167:268. 10.7326/M16-2607 28693043

[21] Li Z Li X Sudfeld CR Liu Y Tang K Huang Y. The effect of the Yingyangbao complementary food supplement on the nutritional status of infants and children: a systematic review and meta-analysis. Nutrients. (2019) 11:2404. 10.3390/nu11102404 31597360 PMC6835467

[22] Victora CG Barros AJ Axelson H Bhutta ZA Chopra M Franca GV. How changes in coverage affect equity in maternal and child health interventions in 35 countdown to 2015 countries: an analysis of national surveys. Lancet. (2012) 380:1149–56. 10.1016/S0140-6736(12)61427-5 22999433

[23] Bradley RH Corwyn RF. Socioeconomic status and child development. Annu Rev Psychol. (2002) 53:371–99. 10.1146/annurev.psych.53.100901.135233 11752490

[24] Rubio-Codina M Grantham-McGregor S. Evolution of the wealth gap in child development and mediating pathways: evidence from a longitudinal study in Bogota, Colombia. Dev Sci. (2019) 22:e12810. 10.1111/desc.12810 30742349

[25] Kendig H Gong CH Yiengprugsawan V Silverstein M Nazroo J. Life course influences on later life health in China: childhood health exposure and socioeconomic mediators during adulthood. SSM Popul Health. (2017) 3:795–802. 10.1016/j.ssmph.2017.10.001 29349264 PMC5769110

[26] Nandi A Glymour MM Kawachi I VanderWeele TJ. Using marginal structural models to estimate the direct effect of adverse childhood social conditions on onset of heart disease, diabetes, and stroke. Epidemiology. (2012) 23:223–32. 10.1097/EDE.0b013e31824570bd 22317806 PMC3414366

[27] Zhang YB Chen C Pan XF Guo J Li Y Franco OH. Associations of healthy lifestyle and socioeconomic status with mortality and incident cardiovascular disease: two prospective cohort studies. BMJ. (2021) 373:n604. 10.1136/bmj.n604 33853828 PMC8044922

[28] Jensen S Xie W Kumar S Haque R Petri WA Nelson CR. Associations of socioeconomic and other environmental factors with early brain development in Bangladeshi infants and children. Dev Cogn Neurosci. (2021) 50:100981. 10.1016/j.dcn.2021.100981 34198217 PMC8254021

[29] Black MM Walker SP Fernald L Andersen CT DiGirolamo AM Lu C. Early childhood development coming of age: science through the life course. Lancet. (2017) 389:77–90. 10.1016/S0140-6736(16)31389-7 27717614 PMC5884058

[30] Moulton V Goodman A Nasim B Ploubidis GB Gambaro L. Parental wealth and children's cognitive ability, mental, and physical health: evidence from the UK Millennium cohort study. Child Dev. (2021) 92:115–23. 10.1111/cdev.13413 32939765

[31] Templin T Cravo Oliveira Hashiguchi T Thomson B Dieleman J Bendavid E. The overweight and obesity transition from the wealthy to the poor in low- and middle-income countries: a survey of household data from 103 countries. PLoS Med. (2019) 16:e1002968. 10.1371/journal.pmed.1002968 31774821 PMC6880978

[32] El-Sayed AM Palma A Freedman LP Kruk ME. Does health insurance mitigate inequities in non-communicable disease treatment? Evidence from 48 low- and middle-income countries. Health Policy. (2015) 119:1164–75. 10.1016/j.healthpol.2015.07.006 26271138 PMC5927367

[33] Ozodiegwu ID Doctor HV Quinn M Mercer LD Omoike OE Mamudu HM. Is the positive association between middle-income and rich household wealth and adult sub-Saharan African women's overweight status modified by the level of education attainment? A cross-sectional study of 22 countries. BMC Public Health. (2020) 20:996. 10.1186/s12889-020-08956-3 32586312 PMC7318408

[34] Conger RD Donnellan MB. An interactionist perspective on the socioeconomic context of human development. Annu Rev Psychol. (2007) 58:175–99. 10.1146/annurev.psych.58.110405.085551 16903807

[35] Walker SP Wachs TD Grantham-McGregor S Black MM Nelson CA Huffman SL. Inequality in early childhood: risk and protective factors for early child development. Lancet. (2011) 378:1325–38. 10.1016/S0140-6736(11)60555-2 21944375

[36] Greene AL Grimsley MD. Age and gender differences in adolescents' preferences for parental advice: mum's the word. J Adolesc Res. (1990) 5:396–413. 10.1177/074355489054002

[37] Zhu Z Sudfeld CR Cheng Y Qi Q Li S Elhoumed M. Anemia and associated factors among adolescent girls and boys at 10–14 years in rural western China. BMC Public Health. (2021) 21:218. 10.1186/s12889-021-10268-z 33499861 PMC7836596

[38] Leve LD Kim HK Pears KC. Childhood temperament and family environment as predictors of internalizing and externalizing trajectories from ages 5 to 17. J Abnorm Child Psychol. (2005) 33:505–20. 10.1007/s10802-005-6734-7 16195947 PMC1468033

[39] Reiss F. Socioeconomic inequalities and mental health problems in children and adolescents: a systematic review. Soc Sci Med. (2013) 90:24–31. 10.1016/j.socscimed.2013.04.026 23746605

[40] Leventhal D Crochemore-Silva I Vidaletti LP Armenta-Paulino N Barros A Victora CG. Delivery channels and socioeconomic inequalities in coverage of reproductive, maternal, newborn, and child health interventions: analysis of 36 cross-sectional surveys in low-income and middle-income countries. Lancet Glob Health. (2021) 9:e1101–9. 10.1016/S2214-109X(21)00204-7 34051180 PMC8295042

