# Supplementary material for: Associations between life-course household wealth mobility and adolescent physical growth, cognitive development and emotional and behavioral problems: A birth cohort in rural western China
Source: Front Public Health. 2023 Feb 2;11:1061251. doi: 10.3389/fpubh.2023.1061251 (PMC9934056; doi:10.3389/fpubh.2023.1061251)
Supplement: Supplementary file 1 [file Table_1.DOCX]

Supplemental material for

**Associations between life-course household wealth mobility and adolescent physical growth, cognitive development and emotional and behavioral problems: A birth cohort in rural western China**

Jiaxin Tian^1†^, Yingze Zhu^1†^, Shuang Liu^2^, Liang Wang^1^, Qi Qi^1^, Qiwei Deng^1^, Amanuel Kidane Andegiorgish^1^, Mohamed Elhoumed^1^, Yue Cheng^3^, Chi Shen^4^, Lingxia Zeng^1,5*^, Zhonghai Zhu^1*^

^†^Co-first authors

**Supplemental Table 1** Associations between household wealth mobility and adolescent WISC-IV test Scores in a birth cohort in rural western China (*n*=1188).

**Supplemental Table 2** Associations between household wealth mobility and adolescent emotional and behavioral problems in a birth cohort in rural western China (*n*=1188).

**Supplemental Table 3** Criteria of model selections for identifying the final trajectories of household wealth from pregnancy to early adolescence.

**Supplemental Table 4** Interaction *P* values between household wealth mobility and parental education and adolescent sex for adolescent HAZ, BAZ, cognitive development, emotional and behavioral problems in a birth cohort in rural western China (*n*=1188).

**Supplemental Table 5** Stratified analysis on associations between household wealth mobility and adolescent HAZ, BAZ, cognitive development, emotional and behavioral problems among mothers with low educational level in a birth cohort in rural western China (*n*=448).

**Supplemental Table 6** Stratified analysis on associations between household wealth mobility and adolescent HAZ, BAZ, cognitive development, emotional and behavioral problems among mothers with high educational level in a birth cohort in rural western China (*n*=737).

**Supplemental Table 7** Stratified analysis on associations between household wealth mobility and adolescent HAZ, BAZ, cognitive development, emotional and behavioral problems among fathers with low educational level in a birth cohort in rural western China (*n*=208).

**Supplemental Table 8** Stratified analysis on associations between household wealth mobility and adolescent HAZ, BAZ, cognitive development, emotional and behavioral problems among fathers with high educational level in a birth cohort in rural western China (*n*=978).

**Supplemental Table 9** Stratified analysis on associations between household wealth mobility and HAZ, BAZ, cognitive development, emotional and behavioral problems among adolescent males in a birth cohort in rural western China (*n*=718).

**Supplemental Table 10** Stratified analysis on associations between household wealth mobility and HAZ, BAZ, cognitive development, emotional and behavioral problems among adolescent females in a birth cohort in rural western China (*n*=480).

**Supplemental Table 11** Associations between household wealth mobility among periods and adolescent HAZ, BAZ, cognitive development, and emotional and behavioral problems from a birth cohort in rural western China after performing inverse probability weighting (*n*=1188).

**Supplemental Table 12** Associations between household wealth mobility and adolescent HAZ, BAZ, cognitive development and emotional and behavioral problems among the lowest 80% household wealth at baseline from a birth cohort in rural western China (*n*=951).

**Supplemental Table 13** Associations between household wealth mobility and adolescent HAZ, BAZ, cognitive development and emotional and behavioral problems among the highest 80% household wealth at baseline from a birth cohort in rural western China (*n*=949).

**Supplemental Table 14** E-values for associations between household wealth mobility and adolescent HAZ, BAZ, cognitive development and emotional and behavioral problems from a birth cohort in rural western China (*n*=1188).

**Supplemental Figure 1** Participant flowchart of a birth cohort in rural western China.

**Supplemental Figure 2** Life-course trajectories of household wealth index (relative-scale mobility) from pregnancy to early adolescence in a birth cohort in rural western China (*n*=1188). Lines show for each trajectory the predicated means of *z* score and 95% confidence limits. We identified four distinct trajectories of household wealth from pregnancy to early adolescence. The Subgroup i, ii, iii and iv were labeled “Consistently low”, “Upward”, “Downward” and “Consistently high”, and the corresponding sample size percentage was presented in the bottom of the figure.

Supplemental Table 1 Associations between household wealth mobility and adolescent WISC-IV test Scores in a birth cohort in rural western China (*n*=1188)

| Household wealth | Scores of WISC-IV test ^a^ | | | |
| --- | --- | --- | --- | --- |
|  | VCI | WMI | PRI | PSI |
| *Household wealth index at single-time point* | | | | |
| Pregnancy/per SD | 0.84(0.07, 1.61) | 0.07(-0.48, 0.63) | 0.38(-0.23, 0.99) | 0.92(0.22, 1.62) |
| Low (Q1) | Ref. | Ref. | Ref. | Ref. |
| Medium (Q2) | 0.16(-1.99, 2.31) | -0.27(-1.82, 1.29) | -0.67(-2.38, 1.04) | 0.91(-1.06, 2.87) |
| High (Q3) | 3.31(0.71, 5.90) | 0.85(-1.03, 2.73) | 1.00(-1.06, 3.06) | 2.42(0.05, 4.79) |
| Mid-childhood per SD | 0.59(-0.04, 1.22) | 0.78(0.33, 1.24) | 1.27(0.77, 1.76) | 1.08(0.50, 1.65) |
| Low (Q1) | Ref. | Ref. | Ref. | Ref. |
| Medium (Q2) | -0.84(-3.00, 1.33) | -0.25(-1.81, 1.30) | 0.30(-1.41, 2.00) | 1.25(-0.72, 3.21) |
| High (Q3) | 1.23(-1.14, 3.61) | 2.54(0.83, 4.25) | 3.74(1.87, 5.61) | 3.30(1.14, 5.46) |
| Early adolescence/per SD | 0.88(0.22, 1.54) | 1.37(0.90, 1.84) | 1.31(0.79, 1.83) | 1.28(0.68, 1.88) |
| Low (Q1) | Ref. | Ref. | Ref. | Ref. |
| Medium (Q2) | 0.20(-1.90, 2.30) | 2.45(0.95, 3.96) | 0.92(-0.73, 2.57) | 0.85(-1.06, 2.75) |
| High (Q3) | 2.96(0.51, 5.41) | 4.25(2.49, 6.01) | 4.57(2.64, 6.50) | 4.94(2.72, 7.17) |
| *Household wealth conditional gains between periods* | | | | |
| Gains from pregnancy to mid-childhood/per SD | 0.48(-0.46, 1.42) | 1.11(0.44, 1.78) | 1.68(0.94, 2.41) | 1.15(0.30, 2.00) |
| Low (Q1) | Ref. | Ref. | Ref. | Ref. |
| Medium (Q2) | 0.31(-1.83, 2.45) | 0.27(-1.27, 1.80) | 0.58(-1.10, 2.27) | 1.53(-0.41, 3.48) |
| High (Q3) | 0.83(-1.42, 3.07) | 2.44(0.83, 4.06) | 3.36(1.59, 5.12) | 2.69(0.65, 4.73) |
| <=0 | Ref. | Ref. | Ref. | Ref. |
| >0 | 0.91(-0.92, 2.75) | 2.32(0.99, 3.64) | 1.94(0.49, 3.39) | 1.40(-0.27, 3.08) |
| Gains from mid-childhood to early adolescence/per SD | 0.64(-0.24, 1.53) | 1.37(0.73, 2.00) | 0.81(0.11, 1.51) | 0.82(0.01, 1.62) |
| Low (Q1) | Ref. | Ref. | Ref. | Ref. |
| Medium (Q2) | 1.16(-0.98, 3.30) | 1.42(-0.12, 2.95) | 0.55(-1.14, 2.25) | -1.23(-3.17, 0.72) |
| High (Q3) | 1.98(-0.22, 4.18) | 3.09(1.51, 4.67) | 2.01(0.27, 3.75) | 1.42(-0.58, 3.42) |
| <=0 | Ref. | Ref. | Ref. | Ref. |
| >0 | 1.17(-0.61, 2.96) | 1.60(0.32, 2.89) | 1.64(0.23, 3.04) | 1.19(-0.43, 2.82) |
| Gains from pregnancy to early adolescence/per SD | 0.84(-0.12, 1.79) | 1.88(1.20, 2.56) | 1.65(0.90, 2.40) | 1.36(0.49, 2.22) |
| Low (Q1) | Ref. | Ref. | Ref. | Ref. |
| Medium (Q2) | -0.34(-2.42, 1.75) | 1.91(0.42, 3.40) | 0.32(-1.32, 1.96) | 0.75(-1.14, 2.64) |
| High (Q3) | 1.69(-0.61, 3.99) | 4.04(2.40, 5.69) | 3.75(1.95, 5.56) | 3.82(1.73, 5.90) |
| <=0 | Ref. | Ref. | Ref. | Ref. |
| >0 | 0.81(-1.00, 2.62) | 2.02(0.72, 3.32) | 2.26(0.83, 3.69) | 1.44(-0.21, 3.09) |
| *Trajectories* | | | | |
| Upward versus Consistently low | 2.28(-0.82, 5.38) | 3.82(1.59, 6.05) | 5.04(2.61, 7.47) | 4.91(2.10, 7.73) |
| Downward versus Consistently low | 1.63(-0.81, 4.07) | 2.30(0.55, 4.06) | 1.61(-0.31, 3.52) | 3.47(1.26, 5.68) |
| Consistently high versus Consistently low | 4.76(1.30, 8.22) | 4.22(1.73, 6.70) | 6.44(3.73, 9.15) | 5.13(2.00, 8.27) |
| Upward versus Consistently high | -2.48(-6.49, 1.52) | -0.40(-3.27, 2.48) | -1.40(-4.54, 1.74) | -0.22(-3.85, 3.41) |
| Downward versus Consistently high | -3.13(-6.54, 0.28) | -1.91(-4.36, 0.53) | -4.83(-7.50, -2.16) | -1.66(-4.75, 1.43) |
| Upward versus Downward | 0.65(-2.75, 4.04) | 1.52(-0.92, 3.96) | 3.43(0.78, 6.09) | 1.44(-1.63, 4.52) |

Abbreviations: WISC-IV, Wechsler Intelligence Scale for Children, Fourth Edition; VCI, verbal comprehension; PRI, perceptual reasoning; WMI, working memory; PSI, processing speed index.

^a^Data are presented with adjusted mean differences and their 95% confidence intervals. The adjustments included parental education, occupation and age, maternal parity and mid-upper arm circumference, randomized regimens by duration, small-for-gestational age, and adolescent sex and age.

Supplemental Table 2 Associations between household wealth mobility and adolescent emotional and behavioral problems in a birth cohort in rural western China (*n*=1188)

| Household wealth | Subscale scores of adolescent emotional and behavioral problems^a^ | | | | | | | |
| --- | --- | --- | --- | --- | --- | --- | --- | --- |
|  | Anxiety/  depression | Withdrawn | Somatic complaints | Social problems | Thought problems | Attention problems | Rule breaking | Aggressive behavior |
| *Household wealth index at single-time point* | | | | | | | | |
| Pregnancy/per SD | -0.02  (-0.22, 0.19) | -0.16  (-0.30, -0.01) | -0.14  (-0.30, 0.02) | -0.20  (-0.37, -0.03) | -0.08  (-0.24, 0.08) | -0.19  (-0.35, -0.04) | -0.26  (-0.42, -0.10) | -0.22  (-0.47, 0.04) |
| Low (Q1) | Ref. | Ref. | Ref. | Ref. | Ref. | Ref. | Ref. | Ref. |
| Medium (Q2) | -0.07  (-0.64, 0.49) | -0.18  (-0.57, 0.22) | -0.22  (-0.65, 0.22) | -0.43  (-0.90, 0.04) | -0.09  (-0.53, 0.34) | -0.37  (-0.80, 0.06) | -0.48  (-0.92, -0.04) | -0.12  (-0.82, 0.57) |
| High (Q3) | -0.35  (-1.03, 0.34) | -0.54  (-1.01, -0.06) | -0.41  (-0.94, 0.12) | -0.69  (-1.26, -0.12) | -0.39  (-0.92, 0.14) | -0.64  (-1.15, -0.12) | -0.96  (-1.50, -0.43) | -0.78  (-1.63, 0.06) |
| Mid-childhood/per SD | 0.04  (-0.13, 0.21) | -0.07  (-0.18, 0.05) | -0.08  (-0.21, 0.05) | -0.10  (-0.24, 0.04) | -0.03  (-0.16, 0.10) | -0.02  (-0.15, 0.11) | -0.05  (-0.18, 0.09) | -0.01  (-0.22, 0.19) |
| Low (Q1) | Ref. | Ref. | Ref. | Ref. | Ref. | Ref. | Ref. | Ref. |
| Medium (Q2) | 0.09  (-0.48, 0.66) | -0.06  (-0.45, 0.34) | 0.03  (-0.41, 0.47) | -0.06  (-0.54, 0.41) | -0.08  (-0.52, 0.36) | -0.18  (-0.62, 0.25) | -0.09  (-0.54, 0.36) | 0.18  (-0.53, 0.88) |
| High (Q3) | 0.14  (-0.49, 0.77) | -0.33  (-0.76, 0.11) | -0.32  (-0.80, 0.17) | -0.35  (-0.88, 0.17) | -0.20  (-0.69, 0.28) | -0.09  (-0.56, 0.39) | -0.09  (-0.59, 0.40) | -0.15  (-0.93, 0.63) |
| Early adolescence/per SD | -0.04  (-0.22, 0.13) | -0.09  (-0.22, 0.03) | -0.14  (-0.28, -0.01) | -0.17  (-0.32, -0.03) | -0.07  (-0.21, 0.06) | -0.03  (-0.16, 0.11) | -0.04  (-0.18, 0.10) | -0.10  (-0.32, 0.11) |
| Low (Q1) | Ref. | Ref. | Ref. | Ref. | Ref. | Ref. | Ref. | Ref. |
| Medium (Q2) | -0.27  (-0.83, 0.28) | -0.35  (-0.74, 0.03) | -0.36  (-0.79, 0.06) | -0.17  (-0.63, 0.29) | -0.23  (-0.66, 0.19) | -0.26  (-0.68, 0.16) | -0.13  (-0.57, 0.31) | 0.09  (-0.59, 0.78) |
| High (Q3) | -0.21  (-0.86, 0.44) | -0.34  (-0.80, 0.11) | -0.57  (-1.08, -0.07) | -0.70  (-1.24, -0.16) | -0.31  (-0.81, 0.19) | -0.03  (-0.52, 0.47) | -0.23  (-0.74, 0.28) | -0.25  (-1.05, 0.56) |
| *Household wealth conditional gains between periods* | | | | | | | | |
| Gains from pregnancy to mid-childhood/per SD | 0.07  (-0.18, 0.31) | -0.03  (-0.20, 0.15) | -0.06  (-0.25, 0.14) | -0.06  (-0.27, 0.15) | -0.01  (-0.20, 0.18) | 0.06  (-0.13, 0.25) | 0.05  (-0.15, 0.25) | 0.08  (-0.23, 0.39) |
| Low (Q1) | Ref. | Ref. | Ref. | Ref. | Ref. | Ref. | Ref. | Ref. |
| Medium (Q2) | -0.11  (-0.68, 0.46) | 0.04  (-0.35, 0.44) | -0.09  (-0.53, 0.35) | -0.18  (-0.66, 0.29) | 0.03  (-0.41, 0.47) | -0.04  (-0.47, 0.39) | -0.12  (-0.57, 0.33) | 0.01  (-0.69, 0.72) |
| High (Q3) | 0.10  (-0.49, 0.70) | -0.08  (-0.49, 0.34) | -0.25  (-0.71, 0.21) | -0.23  (-0.72, 0.27) | -0.12  (-0.57, 0.34) | 0.11  (-0.34, 0.56) | 0.17  (-0.30, 0.64) | 0.07  (-0.66, 0.81) |
| <=0 | Ref. | Ref. | Ref. | Ref. | Ref. | Ref. | Ref. | Ref. |
| >0 | 0.16  (-0.32, 0.65) | 0.09  (-0.25, 0.43) | -0.06  (-0.44, 0.32) | 0.03  (-0.38, 0.43) | -0.21  (-0.59, 0.17) | 0.11  (-0.27, 0.48) | 0.21  (-0.18, 0.59) | 0.23  (-0.37, 0.84) |
| Gains from mid-childhood to early adolescence/per SD | -0.09  (-0.33, 0.14) | -0.05  (-0.21, 0.11) | -0.11  (-0.29, 0.07) | -0.12  (-0.32, 0.07) | -0.06  (-0.24, 0.12) | 0.02  (-0.16, 0.19) | 0.04  (-0.15, 0.22) | -0.09  (-0.38, 0.20) |
| Low (Q1) | Ref. | Ref. | Ref. | Ref. | Ref. | Ref. | Ref. | Ref. |
| Medium (Q2) | 0.14  (-0.42, 0.71) | -0.18  (-0.57, 0.21) | 0.03  (-0.40, 0.47) | 0.06  (-0.41, 0.53) | -0.10  (-0.54, 0.33) | -0.10  (-0.52, 0.33) | 0.11  (-0.34, 0.56) | 0.06  (-0.64, 0.76) |
| High (Q3) | -0.15  (-0.73, 0.43) | -0.02  (-0.42, 0.39) | -0.28  (-0.73, 0.17) | -0.23  (-0.71, 0.26) | -0.13  (-0.57, 0.32) | 0.26  (-0.18, 0.70) | 0.28  (-0.17, 0.74) | 0.05  (-0.67, 0.76) |
| <=0 | Ref. | Ref. | Ref. | Ref. | Ref. | Ref. | Ref. | Ref. |
| >0 | 0.01  (-0.46, 0.49) | -0.08  (-0.40, 0.25) | -0.16  (-0.53, 0.21) | -0.20  (-0.59, 0.20) | -0.16  (-0.53, 0.20) | 0.08  (-0.28, 0.44) | 0.06  (-0.31, 0.43) | -0.21  (-0.79, 0.38) |
| Gains from pregnancy to early adolescence/per SD | -0.05  (-0.30, 0.20) | -0.06  (-0.23, 0.12) | -0.13  (-0.32, 0.07) | -0.14  (-0.36, 0.07) | -0.07  (-0.26, 0.13) | 0.05  (-0.14, 0.24) | 0.07  (-0.13, 0.27) | -0.04  (-0.35, 0.27) |
| Low (Q1) | Ref. | Ref. | Ref. | Ref. | Ref. | Ref. | Ref. | Ref. |
| Medium (Q2) | -0.05  (-0.61, 0.50) | -0.20  (-0.59, 0.18) | -0.31  (-0.74, 0.12) | -0.25  (-0.71, 0.21) | -0.32  (-0.75, 0.10) | -0.18  (-0.59, 0.24) | -0.02  (-0.46, 0.41) | 0.26  (-0.42, 0.95) |
| High (Q3) | -0.21  (-0.82, 0.40) | -0.23  (-0.65, 0.19) | -0.47  (-0.94, 0.0003) | -0.38  (-0.89, 0.13) | -0.22  (-0.69, 0.24) | 0.10  (-0.36, 0.56) | -0.01  (-0.49, 0.47) | -0.26  (-1.01, 0.49) |
| <=0 | Ref. | Ref. | Ref. | Ref. | Ref. | Ref. | Ref. | Ref. |
| >0 | -0.22  (-0.70, 0.26) | -0.19  (-0.52, 0.15) | -0.35  (-0.72, 0.02) | -0.38  (-0.78, 0.02) | -0.23  (-0.60, 0.14) | 0.002  (-0.36, 0.37) | -0.12  (-0.50, 0.26) | -0.36  (-0.96, 0.23) |
| *Trajectories* | | | | | | | | |
| Upward versus Consistently low | 0.05  (-0.78, 0.89) | -0.25  (-0.83, 0.33) | -0.36  (-1.01, 0.28) | -0.68  (-1.38, 0.01) | -0.11  (-0.75, 0.53) | -0.18  (-0.81, 0.45) | 0.11  (-0.54, 0.77) | -0.02  (-1.05, 1.01) |
| Downward versus Consistently low | -0.17  (-0.83, 0.48) | -0.34  (-0.80, 0.11) | -0.23  (-0.74, 0.27) | -0.38  (-0.93, 0.16) | 0.06  (-0.45, 0.56) | -0.15  (-0.65, 0.34) | -0.56  (-1.07, -0.04) | 0.08  (-0.73, 0.89) |
| Consistently high versus Consistently low | -0.01  (-0.94, 0.93) | -0.58  (-1.23, 0.06) | -0.48  (-1.20, 0.24) | -1.06  (-1.84, -0.29) | -0.37  (-1.09, 0.34) | -0.44  (-1.14, 0.27) | -0.70  (-1.43, 0.04) | -0.84  (-1.99, 0.32) |
| Upward versus Consistently high | 0.06  (-1.04, 1.16) | 0.33  (-0.43, 1.09) | 0.12  (-0.73, 0.97) | 0.38  (-0.53, 1.29) | 0.26  (-0.58, 1.11) | 0.25  (-0.58, 1.08) | 0.81  (-0.05, 1.67) | 0.81  (-0.55, 2.17) |
| Downward versus Consistently high | -0.17  (-1.11, 0.77) | 0.24  (-0.41, 0.89) | 0.25  (-0.47, 0.97) | 0.68  (-0.10, 1.46) | 0.43  (-0.29, 1.15) | 0.28  (-0.43, 0.99) | 0.14  (-0.60, 0.88) | 0.91  (-0.25, 2.07) |
| Upward versus Downward | 0.23  (-0.70, 1.15) | 0.09  (-0.55, 0.73) | -0.13  (-0.84, 0.58) | 0.30  (-1.07, 0.47) | -0.17  (-0.88, 0.54) | -0.03  (-0.73, 0.67) | 0.67  (-0.06, 1.39) | -0.10  (-1.24, 1.04) |

^a^Data are presented with adjusted mean differences and their 95% confidence intervals. The adjustments included parental education, occupation and age, maternal parity and mid-upper arm circumference, randomized regimens by duration, small-for-gestational age, and adolescent sex and age.

Supplemental Table 3 Criteria of model selections for identifying the final trajectories of household wealth from pregnancy to early adolescence

| Number of subgroups | Trajectory shapes^a^ | P values for the test of parameters | BIC^b^ | AIC^b^ | APP^c^ | OCC^d^ | Size (%) |
| --- | --- | --- | --- | --- | --- | --- | --- |
| 2 | 1 1 | <0.001/<0.001 | -4526.19 | -4510.95 | 0.95/0.90 | 111/189 | 75.08/24.92 |
| 3 | 1 1 1 | <0.001/0.23/0.007 | -4502.47 | -4479.61 | 0.89/0.74/0.85 | 58/40/243 | 59.62/28.40/11.98 |
| 3 | 1 2 3 | <0.001/0.15/0.99 | -4498.05 | -4467.57 | 0.89/0.74/0.86 | 56/50/261 | 59.17/28.45/12.38 |
| 3 | 2 2 2 | 0.45/0.24/<0.001 | -4497.75 | -4467.27 | 0.89/0.74/0.86 | 56/49/250 | 58.92/28.57/12.51 |
| 3 | 2 2 3 | 0.45/0.24/0.99 | -4501.29 | -4468.27 | 0.89/0.74/0.86 | 56/49/250 | 58.92/28.57/12.51 |
| 3 | 3 3 3 | 0.99/0.99/0.99 | -4508.37 | -4470.27 | 0.89/0.74/0.86 | 56/49/250 | 58.92/28.57/12.51 |
| 4 | 1 1 1 1 | 0.75/0.13/0.09/0.24 | -4506.25 | -4475.77 | 0.70/0.66/0.71/0.81 | 46/17/60/223 | 28.43/41.04/20.53/10.00 |
| 4 | 1 2 3 3 | 0.01/0.03/0.99/0.99 | -4496.57 | -4453.39 | 0.87/0.65/0.65/0.85 | 53/44/91/211 | 55.03/21.17/11.19/12.61 |
| **4** | **2 2 3 2** | **0.26/<0.001/0.99/<0.001** | **-4495.87** | **-4452.69** | **0.86/0.65/0.66/0.84** | **48/90/46/193** | **53.76/11.43/22.22/12.59** |
| 4 | 2 3 3 3 | 0.46/0.99/0.99/0.99 | -4502.95 | -4454.69 | 0.86/0.66/0.65/0.84 | 48/46/90/193 | 53.66/22.27/11.48/12.59 |
| 4 | 3 3 3 3 | 0.99/0.99/0.99/0.99 | -4506..49 | -4455.69 | 0.86/0.66/0.65/0.84 | 48/46/90/193 | 53.66/22.27/11.48/12.59 |
| 5 | 1 1 1 1 1 | 0.32/0.004/<0.001/<0.001/0.02 | -4505.93 | -4467.86 | 0.67/0.80/0.64/0.72/0.83 | 170/27/363/63/264 | 11.36/55.23/3.30/20.44/9.68 |

^a^Trajectory shapes: 1=linear, 2=quadratic, 3=cubic.

^b^BIC, Bayesian information criterion. AIC, Akaike information criterion. The model with the highest (least negative) value of BIC and AIC is preferred.

^c^APP, an average of the posterior probabilities of group membership for individuals assigned to each group, should exceed 0.7, range 0.00-1.00.

^d^OCC, odds of correct classification based on the posterior probabilities of group membership, should exceed 5.

Supplemental Table 4 Interaction *P* values between household wealth mobility and parental education and adolescent sex for adolescent HAZ, BAZ, cognitive development, emotional and behavioral problems in a birth cohort in rural western China (*n*=1188)

| Household wealth | *Z* scores of physical growth | |  | Cognitive development |  | Scores of emotional and behavioral problems | | |
| --- | --- | --- | --- | --- | --- | --- | --- | --- |
|  | HAZ | BAZ |  | FISQ |  | Total problem | Internalizing | Externalizing |
| *Household wealth index at single-time point* | | | | | | | | |
| Pregnancy |  |  |  |  |  |  |  |  |
| P_interaction_^a^ | 0.55 | 0.88 |  | 0.83 |  | 0.22 | 0.43 | 0.13 |
| P_interaction_^b^ | 0.05 | 0.90 |  | 0.42 |  | 0.04 | 0.05 | 0.26 |
| P_interaction_^c^ | 0.49 | 0.41 |  | 0.23 |  | 0.38 | 0.46 | 0.10 |
| Mid-childhood |  |  |  |  |  |  |  |  |
| P_interaction_^a^ | 0.97 | 0.42 |  | 0.36 |  | 0.03 | 0.04 | 0.30 |
| P_interaction_^b^ | 0.09 | 0.15 |  | 0.74 |  | 0.67 | 0.73 | 0.30 |
| P_interaction_^c^ | 0.23 | 0.10 |  | 0.04 |  | 0.36 | 0.26 | 0.19 |
| Early adolescence |  |  |  |  |  |  |  |  |
| P_interaction_^a^ | 0.50 | 0.33 |  | 0.76 |  | 0.32 | 0.81 | 0.94 |
| P_interaction_^b^ | 0.046 | 0.69 |  | 0.42 |  | 0.81 | 0.57 | 0.83 |
| P_interaction_^c^ | 0.30 | 0.01 |  | 0.01 |  | 0.22 | 0.14 | 0.27 |
| *Household wealth conditional gains between periods* | | | | | | | | |
| Gains from pregnancy to mid-childhood |  |  |  |  |  |  |  |  |
| P_interaction_^a^ | 0.90 | 0.45 |  | 0.45 |  | 0.01 | 0.02 | 0.09 |
| P_interaction_^b^ | 0.29 | 0.15 |  | 0.44 |  | 0.23 | 0.70 | 0.15 |
| P_interaction_^c^ | 0.31 | 0.16 |  | 0.10 |  | 0.54 | 0.37 | 0.49 |
| Gains from mid-childhood to early adolescence |  |  |  |  |  |  |  |  |
| P_interaction_^a^ | 0.57 | 0.56 |  | 0.39 |  | 0.99 | 0.41 | 0.77 |
| P_interaction_^b^ | 0.26 | 0.69 |  | 0.28 |  | 0.83 | 0.90 | 0.46 |
| P_interaction_^c^ | 0.62 | 0.04 |  | 0.18 |  | 0.43 | 0.32 | 0.85 |
| Gains from pregnancy to early adolescence |  |  |  |  |  |  |  |  |
| P_interaction_^a^ | 0.68 | 0.36 |  | 0.73 |  | 0.14 | 0.57 | 0.51 |
| P_interaction_^b^ | 0.17 | 0.69 |  | 0.22 |  | 0.67 | 0.90 | 0.91 |
| P_interaction_^c^ | 0.32 | 0.01 |  | 0.06 |  | 0.34 | 0.19 | 0.63 |
| *Trajectories* | | | | | | | | |
| P_interaction_^a^ | 0.91 | 0.29 |  | 0.88 |  | 0.23 | 0.32 | 0.52 |
| P_interaction_^b^ | 0.02 | 0.97 |  | 0.44 |  | 0.16 | 0.03 | 0.44 |
| P_interaction_^c^ | 0.25 | 0.12 |  | 0.049 |  | 0.34 | 0.38 | 0.10 |

Abbreviations: HAZ, height-for- age and sex z score; BAZ, body mass index-for- age and sex *z* score; FSIQ, full-scale intelligent quotient.

^a^*P* values of interaction between household wealth index and maternal education. In addition to interaction term, the models were adjusted for paternal education, parental occupation and age, maternal parity and mid-upper arm circumference, randomized regimens by duration, small-for-gestational age, and adolescent sex and age.

^b^*P* values of interaction between household wealth index and paternal education. In addition to interaction term, the models were adjusted for parental occupation and age, maternal education, parity and mid-upper arm circumference, randomized regimens by duration, small-for-gestational age, and adolescent sex and age.

^c^*P* values of interaction between household wealth index and adolescent sex. In addition to interaction term, the models were adjusted for parental education, occupation and age, maternal parity and mid-upper arm circumference, randomized regimens by duration, small-for-gestational age, and adolescent age.

Supplemental Table 5 Stratified analysis on associations between household wealth mobility and adolescent HAZ, BAZ, cognitive development, emotional and behavioral problems among mothers with low educational level in a birth cohort in rural western China (*n*=448)

| Household wealth | *Z* scores of physical growth^a^ | |  | Cognitive development^a^ |  | Scores of emotional and behavioral problems^a^ | | |
| --- | --- | --- | --- | --- | --- | --- | --- | --- |
|  | HAZ | BAZ |  | FISQ |  | Total problem | Internalizing | Externalizing |
| *Household wealth index at single-time point* | | | | | | | | |
| Pregnancy/per SD | 0.10(0.01, 0.19) | 0.08(-0.02, 0.17) |  | 1.12(0.13, 2.11) |  | -0.43(-2.58, 1.72) | -0.13(-0.79, 0.54) | 0.02(-0.64, 0.68) |
| Low (Q1) | Ref. | Ref. |  | Ref. |  | Ref. | Ref. | Ref. |
| Medium (Q2) | 0.12(-0.10, 0.34) | 0.06(-0.18, 0.29) |  | 0.29(-2.06, 2.65) |  | -1.73(-6.91, 3.45) | -0.10(-1.69, 1.50) | 0.13(-1.46, 1.72) |
| High (Q3) | 0.32(-0.02, 0.65) | 0.26(-0.11, 0.63) |  | 4.57(0.92, 8.21) |  | -0.42(-8.46, 7.62) | -0.49(-2.97, 1.98) | 0.05(-2.41, 2.52) |
| Mid-childhood/ per SD | 0.11(0.03, 0.18) | 0.09(0.01, 0.18) |  | 1.25(0.41, 2.09) |  | -1.68(-3.52, 0.16) | -0.67(-1.23, -0.10) | -0.29(-0.85, 0.28) |
| Low (Q1) | Ref. | Ref. |  | Ref. |  | Ref. | Ref. | Ref. |
| Medium (Q2) | 0.12(-0.10, 0.34) | 0.08(-0.16, 0.33) |  | -0.6(-3.02, 1.83) |  | -1.3(-6.64, 4.04) | -0.39(-2.03, 1.25) | 0.002(-1.63, 1.64) |
| High (Q3) | 0.36(0.09, 0.63) | 0.29(-0.003, 0.59) |  | 2.85(-0.10, 5.81) |  | -2.77(-9.29, 3.76) | -1.65(-3.65, 0.35) | 0.06(-1.94, 2.06) |
| Early adolescence/ per SD | 0.09(0.01, 0.18) | 0.10(0.01, 0.20) |  | 1.41(0.50, 2.32) |  | -1.04(-3.07, 0.99) | -0.32(-0.94, 0.31) | -0.26(-0.88, 0.36) |
| Low (Q1) | Ref. | Ref. |  | Ref. |  | Ref. | Ref. | Ref. |
| Medium (Q2) | 0.14(-0.08, 0.36) | 0.14(-0.10, 0.38) |  | 1.06(-1.31, 3.43) |  | -2.76(-8.00, 2.47) | -0.56(-2.17, 1.06) | -0.56(-2.17, 1.04) |
| High (Q3) | 0.18(-0.13, 0.50) | 0.24(-0.11, 0.58) |  | 4.67(1.25, 8.09) |  | -3.01(-10.61, 4.58) | -0.58(-2.92, 1.75) | -1.11(-3.44, 1.22) |
| *Household wealth conditional gains between periods* | | | | | | | | |
| Gains from pregnancy to mid-childhood/ per SD | 0.02(-0.10, 0.14) | 0.06(-0.07, 0.19) |  | 0.75(-0.52, 2.01) |  | 0.1(-2.68, 2.89) | 0.18(-0.68, 1.04) | -0.15(-1.00, 0.71) |
| Low (Q1) | Ref. | Ref. |  | Ref. |  | Ref. | Ref. | Ref. |
| Medium (Q2) | -0.06(-0.29, 0.17) | 0.07(-0.19, 0.32) |  | -0.05(-2.57, 2.48) |  | -1.68(-7.23, 3.86) | -0.81(-2.51, 0.90) | -0.49(-2.18, 1.21) |
| High (Q3) | 0.23(-0.03, 0.48) | 0.21(-0.07, 0.50) |  | 1.27(-1.52, 4.07) |  | -2.51(-8.64, 3.62) | -1.52(-3.41, 0.36) | -0.04(-1.92, 1.84) |
| <=0 | Ref. | Ref. |  | Ref. |  | Ref. | Ref. | Ref. |
| >0 | 0.28(0.07, 0.49) | 0.12(-0.11, 0.35) |  | 2.52(0.26, 4.79) |  | -2.14(-7.11, 2.83) | -1.06(-2.59, 0.47) | -0.28(-1.80, 1.25) |
| Gains from mid-childhood to early adolescence | 0.02(-0.10, 0.14) | 0.06(-0.07, 0.19) |  | 0.75(-0.52, 2.01) |  | 0.10(-2.68, 2.89) | 0.18(-0.68, 1.04) | -0.15(-1.00, 0.71) |
| Low (Q1) | Ref. | Ref. |  | Ref. |  | Ref. | Ref. | Ref. |
| Medium (Q2) | 0.18(-0.05, 0.40) | 0.11(-0.14, 0.36) |  | 0.32(-2.15, 2.79) |  | 0.14(-5.24, 5.52) | 0.16(-1.49, 1.82) | 0.10(-1.55, 1.74) |
| High (Q3) | 0.01(-0.24, 0.27) | 0.19(-0.09, 0.47) |  | 1.32(-1.51, 4.14) |  | 0.95(-5.28, 7.18) | 0.47(-1.45, 2.38) | 0.20(-1.71, 2.11) |
| <=0 | Ref. | Ref. |  | Ref. |  | Ref. | Ref. | Ref. |
| >0 | -0.02(-0.23, 0.18) | 0.08(-0.15, 0.30) |  | 1.10(-1.13, 3.33) |  | 0.54(-4.36, 5.44) | 0.64(-0.87, 2.15) | -0.06(-1.56, 1.44) |
| Gains from pregnancy to early adolescence | 0.08(-0.04, 0.21) | 0.11(-0.03, 0.25) |  | 1.43(0.07, 2.79) |  | -1.26(-4.26, 1.74) | -0.39(-1.32, 0.53) | -0.39(-1.31, 0.52) |
| Low (Q1) | Ref. | Ref. |  | Ref. |  | Ref. | Ref. | Ref. |
| Medium (Q2) | 0.01(-0.21, 0.23) | 0.08(-0.16, 0.32) |  | 0.96(-1.47, 3.39) |  | -0.92(-6.27, 4.43) | 0.07(-1.58, 1.71) | -0.25(-1.89, 1.39) |
| High (Q3) | 0.24(-0.04, 0.52) | 0.26(-0.04, 0.57) |  | 3.06(0.02, 6.10) |  | -2.59(-9.31, 4.12) | -0.86(-2.93, 1.20) | -0.56(-2.62, 1.50) |
| <=0 | Ref. | Ref. |  | Ref. |  | Ref. | Ref. | Ref. |
| >0 | 0.06(-0.14, 0.27) | 0.13(-0.09, 0.36) |  | 1.48(-0.77, 3.72) |  | -3.06(-8.01, 1.90) | -0.72(-2.25, 0.81) | -1.05(-2.57, 0.47) |
| *Trajectories* | | | | | | | | |
| Upward versus Consistently low | 0.12(-0.26, 0.51) | 0.29(-0.14, 0.72) |  | 5.1(0.90, 9.31) |  | -7.99(-17.42, 1.44) | -2.94(-5.83, -0.05) | -2.56(-5.45, 0.32) |
| Downward versus Consistently low | 0.24(-0.08, 0.56) | -0.02(-0.38, 0.34) |  | 4.06(0.58, 7.54) |  | -2.93(-10.75, 4.90) | -1.51(-3.91, 0.89) | 0.19(-2.21, 2.58) |
| Consistently high versus Consistently low | 0.43(-0.14, 1.00) | 0.56(-0.06, 1.19) |  | 6.10(-0.28, 12.47) |  | -1.97(-15.44, 11.49) | -1.26(-5.39, 2.87) | -0.78(-4.90, 3.35) |
| Upward versus Consistently high | -0.3(-0.96, 0.35) | -0.27(-0.99, 0.45) |  | -1.00(-8.30, 6.31) |  | -6.02(-21.57, 9.54) | -1.68(-6.45, 3.09) | -1.79(-6.55, 2.97) |
| Downward versus Consistently high | -0.19(-0.82, 0.43) | -0.59(-1.27, 0.10) |  | -2.04(-8.98, 4.90) |  | -0.96(-15.76, 13.85) | -0.25(-4.79, 4.29) | 0.96(-3.57, 5.50) |
| Upward versus Downward | -0.14(-0.60, 0.32) | 0.33(-0.23, 0.89) |  | 1.49(-3.60, 6.58) |  | -5.86(-18.11, 6.38) | -1.84(-5.43, 1.75) | -2.84(-6.35, 0.68) |

Abbreviations: HAZ, height-for- age and sex z score; BAZ, body mass index-for- age and sex z score; FSIQ, full-scale intelligent quotient.

^a^Data are presented with adjusted mean differences and their 95% confidence intervals. The adjustments included parental occupation and age, paternal education, maternal parity and mid-upper arm circumference, randomized regimens by duration, small-for-gestational age, and adolescent sex and age.

Supplemental Table 6 Stratified analysis on associations between household wealth mobility and adolescent HAZ, BAZ, cognitive development, emotional and behavioral problems among mothers with high educational level in a birth cohort in rural western China (*n*=737)

| Household wealth | *Z* scores of physical growth^a^ | |  | Cognitive development^a^ |  | Scores of emotional and behavioral problems^a^ | | |
| --- | --- | --- | --- | --- | --- | --- | --- | --- |
|  | HAZ | BAZ |  | FISQ |  | Total problem | Internalizing | Externalizing |
| *Household wealth index at single-time point* | | | | | | | | |
| Pregnancy/per SD | 0.07(0.003, 0.14) | -0.01(-0.08, 0.07) |  | 0.61(-0.15, 1.38) |  | -2.35(-4.01, -0.68) | -0.44(-0.99, 0.11) | -0.82(-1.29, -0.36) |
| Low (Q1) | Ref. | Ref. |  | Ref. |  | Ref. | Ref. | Ref. |
| Medium (Q2) | -0.02(-0.24, 0.19) | -0.10(-0.34, 0.14) |  | -0.08(-2.49, 2.32) |  | -4.55(-9.65, 0.55) | -0.90(-2.59, 0.79) | -1.43(-2.86, -0.003) |
| High (Q3) | 0.14(-0.09, 0.37) | -0.001(-0.26, 0.26) |  | 1.86(-0.73, 4.44) |  | -7.96(-13.46, -2.46) | -1.84(-3.66, -0.02) | -2.92(-4.46, -1.38) |
| Mid-childhood/ per SD | 0.09(0.04, 0.15) | -0.01(-0.07, 0.05) |  | 1.24(0.64, 1.84) |  | 0.94(-0.37, 2.25) | 0.19(-0.24, 0.62) | 0.06(-0.31, 0.43) |
| Low (Q1) | Ref. | Ref. |  | Ref. |  | Ref. | Ref. | Ref. |
| Medium (Q2) | 0.19(-0.02, 0.39) | 0.08(-0.16, 0.31) |  | 0.99(-1.34, 3.31) |  | 1.92(-3.14, 6.98) | 0.49(-1.17, 2.16) | 0.06(-1.36, 1.48) |
| High (Q3) | 0.38(0.17, 0.60) | -0.06(-0.30, 0.18) |  | 4.08(1.68, 6.47) |  | 2.02(-3.21, 7.25) | 0.32(-1.40, 2.05) | -0.32(-1.79, 1.15) |
| Early adolescence/ per SD | 0.10(0.05, 0.16) | 0.03(-0.04, 0.09) |  | 1.63(1.02, 2.25) |  | -0.15(-1.49, 1.19) | -0.24(-0.68, 0.20) | -0.12(-0.50, 0.26) |
| Low (Q1) | Ref. | Ref. |  | Ref. |  | Ref. | Ref. | Ref. |
| Medium (Q2) | 0.27(0.07, 0.47) | 0.30(0.07, 0.53) |  | 1.53(-0.70, 3.76) |  | -1.53(-6.39, 3.34) | -1.33(-2.93, 0.27) | 0.44(-0.92, 1.81) |
| High (Q3) | 0.38(0.16, 0.59) | 0.09(-0.14, 0.33) |  | 5.90(3.56, 8.25) |  | -0.86(-5.97, 4.25) | -1.45(-3.12, 0.23) | -0.20(-1.64, 1.23) |
| *Household wealth conditional gains between periods* | | | | | | | | |
| Gains from pregnancy to mid-childhood/ per SD | 0.10(0.02, 0.18) | -0.01(-0.10, 0.08) |  | 1.55(0.65, 2.44) |  | 2.34(0.40, 4.28) | 0.46(-0.18, 1.10) | 0.42(-0.12, 0.97) |
| Low (Q1) | Ref. | Ref. |  | Ref. |  | Ref. | Ref. | Ref. |
| Medium (Q2) | 0.13(-0.07, 0.33) | 0.07(-0.15, 0.30) |  | 1.58(-0.64, 3.80) |  | 2.12(-2.78, 7.01) | 0.24(-1.37, 1.85) | 0.21(-1.17, 1.59) |
| High (Q3) | 0.21(0.01, 0.41) | -0.005(-0.23, 0.22) |  | 3.93(1.69, 6.16) |  | 4.15(-0.73, 9.03) | 0.79(-0.82, 2.40) | 0.56(-0.81, 1.94) |
| <=0 | Ref. | Ref. |  | Ref. |  | Ref. | Ref. | Ref. |
| >0 | 0.21(0.04, 0.37) | 0.01(-0.18, 0.19) |  | 1.78(-0.07, 3.63) |  | 4.54(0.50, 8.57) | 1.08(-0.25, 2.41) | 0.96(-0.18, 2.09) |
| Gains from mid-childhood to early adolescence/per SD | 0.06(-0.01, 0.14) | 0.05(-0.04, 0.13) |  | 1.31(0.48, 2.14) |  | -0.60(-2.39, 1.18) | -0.44(-1.02, 0.15) | -0.10(-0.60, 0.40) |
| Low (Q1) | Ref. | Ref. |  | Ref. |  | Ref. | Ref. | Ref. |
| Medium (Q2) | 0.16(-0.05, 0.36) | 0.40(0.17, 0.63) |  | 1.32(-0.95, 3.58) |  | 0.34(-4.60, 5.27) | -0.25(-1.87, 1.37) | 0.07(-1.32, 1.45) |
| High (Q3) | 0.25(0.05, 0.44) | 0.20(-0.02, 0.42) |  | 3.62(1.44, 5.81) |  | -0.30(-5.01, 4.40) | -0.94(-2.48, 0.61) | 0.24(-1.08, 1.56) |
| <=0 | Ref. | Ref. |  | Ref. |  | Ref. | Ref. | Ref. |
| >0 | 0.16(0.001, 0.32) | 0.13(-0.04, 0.31) |  | 2.18(0.39, 3.97) |  | -1.04(-4.94, 2.86) | -0.74(-2.02, 0.54) | -0.25(-1.35, 0.84) |
| Gains from pregnancy to early adolescence/per SD | 0.11(0.03, 0.19) | 0.04(-0.05, 0.13) |  | 1.99(1.11, 2.87) |  | 0.75(-1.17, 2.66) | -0.15(-0.79, 0.48) | 0.17(-0.37, 0.71) |
| Low (Q1) | Ref. | Ref. |  | Ref. |  | Ref. | Ref. | Ref. |
| Medium (Q2) | 0.22(0.02, 0.42) | 0.30(0.08, 0.52) |  | 0.74(-1.44, 2.92) |  | -1.05(-5.84, 3.74) | -1.06(-2.63, 0.52) | 0.62(-0.73, 1.96) |
| High (Q3) | 0.32(0.12, 0.52) | 0.13(-0.10, 0.35) |  | 4.54(2.31, 6.77) |  | 0.70(-4.15, 5.55) | -0.90(-2.49, 0.70) | -0.03(-1.39, 1.34) |
| <=0 | Ref. | Ref. |  | Ref. |  | Ref. | Ref. | Ref. |
| >0 | 0.20(0.04, 0.36) | 0.10(-0.08, 0.28) |  | 2.39(0.58, 4.21) |  | -0.02(-3.98, 3.94) | -0.79(-2.09, 0.52) | -0.19(-1.31, 0.92) |
| *Trajectories* | | | | | | | | |
| Upward versus Consistently low | 0.27(0.0003, 0.53) | -0.15(-0.45, 0.14) |  | 5.36(2.41, 8.31) |  | 4.31(-2.23, 10.85) | 0.63(-1.52, 2.79) | 1.22(-0.62, 3.05) |
| Downward versus Consistently low | 0.14(-0.06, 0.35) | -0.13(-0.36, 0.11) |  | 2.34(0.05, 4.63) |  | -0.94(-6.01, 4.14) | -0.45(-2.13, 1.22) | -0.80(-2.22, 0.62) |
| Consistently high versus Consistently low | 0.42(0.15, 0.70) | -0.10(-0.40, 0.21) |  | 7.20(4.15, 10.24) |  | -3.28(-10.11, 3.55) | -0.70(-2.95, 1.56) | -1.82(-3.73, 0.09) |
| Upward versus Consistently high | -0.15(-0.48, 0.17) | -0.06(-0.42, 0.30) |  | -1.83(-5.38, 1.72) |  | 7.59(-0.55, 15.73) | 1.33(-1.36, 4.01) | 3.04(0.76, 5.31) |
| Downward versus Consistently high | -0.28(-0.54, -0.02) | -0.03(-0.32, 0.26) |  | -4.86(-7.72, -2.00) |  | 2.34(-4.23, 8.92) | 0.24(-1.93, 2.41) | 1.02(-0.82, 2.86) |
| Upward versus Downward | 0.13(-0.15, 0.42) | -0.03(-0.38, 0.32) |  | 2.63(-0.49, 5.75) |  | 5.17(-1.87, 12.21) | 1.05(-1.35, 3.45) | 1.98(-0.07, 4.03) |

Abbreviations: HAZ, height-for- age and sex z score; BAZ, body mass index-for- age and sex z score; FSIQ, full-scale intelligent quotient.

^a^Data are presented with adjusted mean differences and their 95% confidence intervals. The adjustments include parental occupation and age, paternal education, maternal parity and mid-upper arm circumference, randomized regimens by duration, small-for-gestational age, and adolescent sex and age.

Supplemental Table 7 Stratified analysis on associations between household wealth mobility and adolescent HAZ, BAZ, cognitive development, emotional and behavioral problems among fathers with low educational level in a birth cohort in rural western China (*n*=208)

| Household wealth | *Z* scores of physical growth^a^ | |  | Cognitive development^a^ |  | Scores of emotional and behavioral problems^a^ | | |
| --- | --- | --- | --- | --- | --- | --- | --- | --- |
|  | HAZ | BAZ |  | FISQ |  | Total problem | Internalizing | Externalizing |
| *Household wealth index at single-time point* | | | | | | | | |
| Pregnancy/per SD | 0.06(-0.07, 0.19) | 0.06(-0.08, 0.20) |  | 1.04(-0.39, 2.47) |  | -2.00(-5.26, 1.27) | -0.51(-1.52, 0.49) | -0.60(-1.59, 0.40) |
| Low (Q1) | Ref. | Ref. |  | Ref. |  | Ref. | Ref. | Ref. |
| Medium (Q2) | 0.26(-0.05, 0.56) | 0.10(-0.24, 0.43) |  | -0.10(-3.59, 3.39) |  | -5.89(-13.84, 2.06) | -1.04(-3.49, 1.41) | -0.79(-3.19, 1.68) |
| High (Q3) | 0.05(-0.47, 0.57) | 0.29(-0.29, 0.87) |  | 3.29(-2.68, 9.27) |  | -8.12(-21.87, 5.62) | -2.49(-6.72, 1.75) | -1.70(-5.91, 2.51) |
| Mid-childhood/per SD | 0.09(-0.02, 0.21) | 0.06(-0.07, 0.19) |  | 1.00(-0.31, 2.31) |  | -2.04(-5.05, 0.97) | -0.45(-1.38, 0.48) | -0.52(-1.44, 0.40) |
| Low (Q1) | Ref. | Ref. |  | Ref. |  | Ref. | Ref. | Ref. |
| Medium (Q2) | 0.32(0.02, 0.62) | 0.14(-0.20, 0.47) |  | 0.23(-3.18, 3.65) |  | -1.85(-9.68, 5.98) | 0.23(-2.17, 2.64) | -0.41(-2.80, 1.98) |
| High (Q3) | 0.24(-0.18, 0.66) | 0.26(-0.20, 0.73) |  | 4.70(-0.08, 9.48) |  | -8.01(-19.06, 3.04) | -2.00(-5.40, 1.40) | -1.97(-5.34, 1.40) |
| Early adolescence/per SD | 0.07(-0.05, 0.19) | 0.06(-0.07, 0.19) |  | 1.87(0.54, 3.19) |  | -1.18(-4.29, 1.93) | -0.25(-1.21, 0.71) | -0.37(-1.31, 0.58) |
| Low (Q1) | Ref. | Ref. |  | Ref. |  | Ref. | Ref. | Ref. |
| Medium (Q2) | 0.32(0.01, 0.63) | 0.001(-0.35, 0.35) |  | 3.81(0.27, 7.35) |  | -3.32(-11.49, 4.85) | -1.51(-4.01, 0.99) | -0.82(-3.30, 1.67) |
| High (Q3) | -0.001(-0.43, 0.43) | 0.14(-0.34, 0.62) |  | 3.88(-1.03, 8.79) |  | -3.27(-14.79, 8.25) | 0.10(-3.43, 3.62) | -1.71(-5.21, 1.80) |
| *Household wealth conditional gains between periods* | | | | | | | | |
| Gains from pregnancy to mid-childhood/per SD | 0.10(-0.07, 0.27) | 0.05(-0.14, 0.24) |  | 0.85(-1.08, 2.79) |  | -1.80(-6.24, 2.64) | -0.36(-1.72, 1.01) | -0.41(-1.77, 0.94) |
| Low (Q1) | Ref. | Ref. |  | Ref. |  | Ref. | Ref. | Ref. |
| Medium (Q2) | 0.02(-0.30, 0.34) | 0.16(-0.19, 0.52) |  | 0.62(-3.03, 4.27) |  | -1.07(-9.46, 7.32) | 0.36(-2.21, 2.94) | -1.11(-3.66, 1.44) |
| High (Q3) | 0.06(-0.31, 0.43) | 0.11(-0.30, 0.52) |  | 1.96(-2.25, 6.16) |  | -1.14(-10.84, 8.56) | -0.06(-3.03, 2.92) | -0.11(-3.05, 2.84) |
| <=0 | Ref. | Ref. |  | Ref. |  | Ref. | Ref. | Ref. |
| >0 | 0.16(-0.13, 0.45) | -0.04(-0.35, 0.28) |  | 1.22(-2.06, 4.51) |  | -1.68(-9.18, 5.81) | -0.21(-2.52, 2.09) | -0.21(-2.49, 2.08) |
| Gains from mid-childhood to early adolescence/per SD | 0.01(-0.15, 0.18) | 0.03(-0.15, 0.22) |  | 1.90(0.02, 3.79) |  | 0.43(-3.90, 4.76) | 0.13(-1.20, 1.46) | 0.03(-1.28, 1.35) |
| Low (Q1) | Ref. | Ref. |  | Ref. |  | Ref. | Ref. | Ref. |
| Medium (Q2) | 0.16(-0.16, 0.47) | 0.12(-0.23, 0.46) |  | -0.64(-4.18, 2.91) |  | 1.70(-6.49, 9.89) | 0.35(-2.17, 2.86) | 0.82(-1.67, 3.31) |
| High (Q3) | -0.02(-0.39, 0.35) | -0.04(-0.46, 0.37) |  | 3.42(-0.79, 7.64) |  | 1.42(-8.25, 11.09) | 0.16(-2.81, 3.13) | 0.14(-2.80, 3.08) |
| <=0 | Ref. | Ref. |  | Ref. |  | Ref. | Ref. | Ref. |
| >0 | 0.05(-0.24, 0.34) | -0.01(-0.33, 0.31) |  | 3.73(0.46, 7.01) |  | 0.72(-6.91, 8.34) | 0.42(-1.92, 2.76) | -0.53(-2.85, 1.79) |
| Gains from pregnancy to early adolescence/per SD | 0.06(-0.11, 0.24) | 0.05(-0.14, 0.24) |  | 2.07(0.12, 4.01) |  | -0.51(-5.05, 4.03) | -0.06(-1.45, 1.34) | -0.17(-1.55, 1.21) |
| Low (Q1) | Ref. | Ref. |  | Ref. |  | Ref. | Ref. | Ref. |
| Medium (Q2) | -0.02(-0.34, 0.30) | -0.04(-0.39, 0.32) |  | 1.63(-2.00, 5.25) |  | -0.44(-8.73, 7.85) | 0.46(-2.09, 3.00) | 0.22(-2.31, 2.74) |
| High (Q3) | 0.22(-0.15, 0.59) | 0.08(-0.33, 0.49) |  | 3.20(-1.02, 7.42) |  | -1.29(-11.05, 8.47) | -0.38(-3.37, 2.62) | -0.69(-3.66, 2.28) |
| <=0 | Ref. | Ref. |  | Ref. |  | Ref. | Ref. | Ref. |
| >0 | 0.11(-0.17, 0.40) | 0.04(-0.27, 0.36) |  | 3.23(0.003, 6.46) |  | -4.45(-11.88, 2.98) | -1.09(-3.37, 1.20) | -2.02(-4.27, 0.23) |
| *Trajectories* | | | | | | | | |
| Upward versus Consistently low | -0.11(-0.67, 0.46) | 0.08(-0.54, 0.70) |  | 3.09(-3.32, 9.50) |  | -5.90(-21.08, 9.28) | -1.41(-6.08, 3.25) | -1.55(-6.17, 3.08) |
| Downward versus Consistently low | 0.15(-0.41, 0.72) | 0.07(-0.56, 0.69) |  | 1.28(-5.15, 7.71) |  | -1.90(-16.83, 13.03) | -0.59(-5.18, 4.00) | -0.12(-4.67, 4.42) |
| Consistently high versus Consistently low | 0.36(-0.66, 1.38) | 0.17(-0.95, 1.30) |  | 2.10(-9.52, 13.72) |  | -10.63(-36.83, 15.57) | -3.01(-11.07, 5.04) | -3.43(-11.41, 4.55) |
| Upward versus Consistently high | -0.47(-1.59, 0.65) | -0.10(-1.34, 1.14) |  | 0.99(-11.81, 13.79) |  | 4.73(-24.49, 33.95) | 1.60(-7.38, 10.58) | 1.88(-7.02, 10.78) |
| Downward versus Consistently high | -0.21(-1.31, 0.90) | -0.11(-1.32, 1.11) |  | -0.82(-13.39, 11.75) |  | 8.73(-19.67, 37.12) | 2.43(-6.30, 11.16) | 3.31(-5.34, 11.96) |
| Upward versus Downward | -0.26(-1.02, 0.49) | 0.01(-0.82, 0.84) |  | 1.81(-6.79, 10.40) |  | -4.00(-24.46, 16.47) | -0.83(-7.12, 5.46) | -1.42(-7.66, 4.81) |

Abbreviations: HAZ, height-for- age and sex z score; BAZ, body mass index-for- age and sex z score; FSIQ, full-scale intelligent quotient.

^a^Data are presented with adjusted mean differences and their 95% confidence intervals. The adjustments include parental occupation and age, maternal education, maternal parity and mid-upper arm circumference, randomized regimens by duration, small-for-gestational age, and adolescent sex and age.

Supplemental Table 8 Stratified analysis on associations between household wealth mobility and adolescent HAZ, BAZ, cognitive development, emotional and behavioral problems among fathers with high educational level in a birth cohort in rural western China (*n*=978)

| Household wealth | *Z* scores of physical growth^a^ | |  | Cognitive development^a^ |  | Scores of emotional and behavioral problems^a^ | | |
| --- | --- | --- | --- | --- | --- | --- | --- | --- |
|  | HAZ | BAZ |  | FISQ |  | Total problem | Internalizing | Externalizing |
| *Household wealth index at single-time point* | | | | | | | | |
| Pregnancy/per SD | 0.09(0.03, 0.15) | 0.03(-0.04, 0.09) |  | 0.75(0.08, 1.42) |  | -1.70(-3.12, -0.28) | -0.36(-0.82, 0.10) | -0.54(-0.95, -0.13) |
| Low (Q1) | Ref. | Ref. |  | Ref. |  | Ref. | Ref. | Ref. |
| Medium (Q2) | -0.001(-0.18, 0.17) | -0.04(-0.23, 0.16) |  | 0.15(-1.79, 2.10) |  | -2.57(-6.65, 1.51) | -0.39(-1.71, 0.94) | -0.63(-1.81, 0.56) |
| High (Q3) | 0.22(0.02, 0.42) | 0.09(-0.14, 0.31) |  | 2.61(0.39, 4.84) |  | -5.02(-9.71, -0.33) | -1.30(-2.82, 0.22) | -1.91(-3.26, -0.54) |
| Mid-childhood/per SD | 0.10(0.05, 0.15) | 0.03(-0.02, 0.09) |  | 1.29(0.76, 1.82) |  | 0.13(-1.01, 1.27) | -0.15(-0.52, 0.22) | -0.03(-0.36, 0.31) |
| Low (Q1) | Ref. | Ref. |  | Ref. |  | Ref. | Ref. | Ref. |
| Medium (Q2) | 0.12(-0.05, 0.29) | 0.09(-0.11, 0.28) |  | 0.31(-1.61, 2.24) |  | 0.88(-3.24, 5.00) | 0.04(-1.29, 1.38) | 0.13(-1.06, 1.33) |
| High (Q3) | 0.38(0.19, 0.56) | 0.06(-0.14, 0.27) |  | 3.74(1.71, 5.77) |  | -0.04(-4.39, 4.32) | -0.68(-2.09, 0.73) | -0.23(-1.50, 1.03) |
| Early adolescence/per SD | 0.11(0.06, 0.17) | 0.06(0.004, 0.12) |  | 1.53(0.98, 2.09) |  | -0.50(-1.70, 0.70) | -0.36(-0.75, 0.03) | -0.12(-0.47, 0.22) |
| Low (Q1) | Ref. | Ref. |  | Ref. |  | Ref. | Ref. | Ref. |
| Medium (Q2) | 0.21(0.04, 0.38) | 0.33(0.14, 0.51) |  | 0.96(-0.88, 2.80) |  | -2.20(-6.16, 1.75) | -1.03(-2.31, 0.24) | 0.14(-1.01, 1.29) |
| High (Q3) | 0.38(0.19, 0.57) | 0.18(-0.03, 0.39) |  | 5.70(3.62, 7.79) |  | -2.10(-6.61, 2.41) | -1.63(-3.09, -0.18) | -0.38(-1.69, 0.93) |
| *Household wealth conditional gains between periods* | | | | | | | | |
| Gains from pregnancy to mid-childhood/per SD | 0.11(0.04, 0.18) | 0.04(-0.05, 0.12) |  | 1.56(0.77, 2.35) |  | 0.94(-0.76, 2.64) | -0.06(-0.61, 0.49) | 0.20(-0.29, 0.70) |
| Low (Q1) | Ref. | Ref. |  | Ref. |  | Ref. | Ref. | Ref. |
| Medium (Q2) | 0.04(-0.13, 0.21) | 0.04(-0.15, 0.22) |  | 1.02(-0.86, 2.89) |  | 0.77(-3.27, 4.82) | -0.31(-1.62, 1.00) | 0.13(-1.05, 1.30) |
| High (Q3) | 0.23(0.06, 0.41) | 0.08(-0.12, 0.27) |  | 3.25(1.31, 5.18) |  | 0.96(-3.19, 5.11) | -0.48(-1.82, 0.87) | 022(-0.99, 1.43) |
| <=0 | Ref. | Ref. |  | Ref. |  | Ref. | Ref. | Ref. |
| >0 | 0.25(0.11, 0.40) | 0.09(-0.07, 0.25) |  | 2.23(0.63, 3.84) |  | 1.81(-1.64, 5.26) | 0.06(-1.06, 1.18) | 0.49(-0.51, 1.49) |
| Gains from mid-childhood to early adolescence/per SD | 0.07(-0.002, 0.13) | 0.06(-0.02, 0.13) |  | 1.03(0.28, 1.77) |  | -0.50(-2.09, 1.09) | -0.33(-0.85, 0.18) | -0.50(-0.51, 0.41) |
| Low (Q1) | Ref. | Ref. |  | Ref. |  | Ref. | Ref. | Ref. |
| Medium (Q2) | 0.20(0.03, 0.37) | 0.35(0.16, 0.54) |  | 0.77(-1.12, 2.67) |  | 0.16(-3.88, 4.20) | -0.24(-1.54, 1.07) | 0.13(-1.04, 1.31) |
| High (Q3) | 0.23(0.05, 0.40) | 0.24(0.05, 0.43) |  | 2.74(0.84, 4.64) |  | -0.42(-4.46, 3.61) | -0.69(-2.00, 0.62) | 0.32(-0.85, 1.49) |
| <=0 | Ref. | Ref. |  | Ref. |  | Ref. | Ref. | Ref. |
| >0 | 0.12(-0.02, 0.26) | 0.16(0.002, 0.31) |  | 1.52(-0.04, 3.07) |  | -1.00(-4.31, 2.32) | -0.51(-1.58, 0.57) | -0.09(-1.06, 0.87) |
| Gains from pregnancy to early adolescence/per SD | 0.12(0.05, 0.19) | 0.07(-0.01, 0.15) |  | 1.80(0.99, 2.61) |  | 0.07(-1.66, 1.80) | -0.34(-0.90, 0.22) | 0.07(-0.43, 0.58) |
| Low (Q1) | Ref. | Ref. |  | Ref. |  | Ref. | Ref. | Ref. |
| Medium (Q2) | 0.20(0.03, 0.36) | 0.30(0.12, 0.49) |  | 0.55(-1.27, 2.37) |  | -1.67(-5.60, 2.26) | -1.03(-2.30, 0.24) | 0.23(-0.91, 1.37) |
| High (Q3) | 0.32(0.14, 0.50) | 0.21(0.01, 0.41) |  | 4.46(2.47, 6.44) |  | -1.17(-5.44, 3.10) | -1.28(-2.66, 0.10) | -0.23(-1.47, 1.01) |
| <=0 | Ref. | Ref. |  | Ref. |  | Ref. | Ref. | Ref. |
| >0 | 0.17(0.03, 0.32) | 0.15(-0.01, 0.31) |  | 2.00(0.42, 3.58) |  | -1.04(-4.43, 2.34) | -0.94(-2.03, 0.16) | -0.20(-1.18, 0.79) |
| *Trajectories* | | | | | | | | |
| Upward versus Consistently low | 0.29(0.05, 0.53) | 0.0002(-0.27, 0.27) |  | 5.63(3.02, 8.24) |  | 0.51(-5.20, 6.22) | -0.62(-2.47, 1.23) | 0.13(-1.53, 1.78) |
| Downward versus Consistently low | 0.21(0.03, 0.40) | -0.04(-0.24, 0.17) |  | 2.97(0.96, 4.98) |  | -1.44(-5.84, 2.97) | -0.86(-2.29, 0.56) | -0.47(-1.75, 0.80) |
| Consistently high versus Consistently low | 0.49(0.24, 0.74) | 0.10(-0.18, 0.38) |  | 7.31(4.51, 10.11) |  | -3.41(-9.56, 2.74) | -1.32(-3.31, 0.68) | -1.44(-3.23, 0.34) |
| Upward versus Consistently high | -0.20(-0.50, 0.09) | -0.10(-0.43, 0.23) |  | -1.68(-4.94, 1.57) |  | 3.92(-3.35, 11.19) | 0.69(-1.66, 3.05) | 1.57(-0.54, 3.68) |
| Downward versus Consistently high | -0.28(-0.52, -0.03) | -0.14(-0.42, 0.14) |  | -4.34(-7.07, -1.62) |  | 1.98(-4.14, 8.09) | 0.45(-1.53, 2.43) | 0.97(-0.81, 2.74) |
| Upward versus Downward | 0.07(-0.18, 0.33) | 0.04(-0.25, 0.32) |  | 2.66(-0.13, 5.46) |  | 1.95(-4.25, 8.14) | 0.24(-1.76, 2.25) | 0.60(-1.20, 2.40) |

Abbreviations: HAZ, height-for- age and sex z score; BAZ, body mass index-for- age and sex z score; FSIQ, full-scale intelligent quotient.

^a^Data are presented with adjusted mean differences and their 95% confidence intervals. The adjustments include parental occupation and age, maternal education, maternal parity and mid-upper arm circumference, randomized regimens by duration, small-for-gestational age, and adolescent sex and age.

Supplemental Table 9 Stratified analysis on associations between household wealth mobility and HAZ, BAZ, cognitive development, emotional and behavioral problems among adolescent males in a birth cohort in rural western China (*n*=718)

| Household wealth | *Z* scores of physical growth^a^ | |  | Cognitive development^a^ |  | Scores of emotional and behavioral problems^a^ | | |
| --- | --- | --- | --- | --- | --- | --- | --- | --- |
|  | HAZ | BAZ |  | FISQ |  | Total problem | Internalizing | Externalizing |
| *Household wealth index at single-time point* | | | | | | | | |
| Pregnancy/per SD | 0.10(0.03, 0.17) | 0.03(-0.06, 0.11) |  | 0.68(-0.07, 1.43) |  | -2.41(-4.21, -0.62) | -0.55(-1.10, 0.005) | -0.82(-1.36, -0.27) |
| Low (Q1) | Ref. | Ref. |  | Ref. |  | Ref. | Ref. | Ref. |
| Medium (Q2) | 0.11(-0.09, 0.31) | -0.01(-0.24, 0.21) |  | -0.08(-2.14, 1.97) |  | -4.90(-9.75, -0.05) | -1.00(-2.48, 0.49) | -0.95(-2.43, 0.53) |
| High (Q3) | 0.24(-0.001, 0.49) | 0.13(-0.15, 0.41) |  | 2.30(-0.24, 4.84) |  | -7.76(-13.76, -1.77) | -2.04(-3.88, -0.20) | -2.70(-4.53, -0.88) |
| Mid-childhood/per SD | 0.11(0.05, 0.17) | 0.05(-0.02, 0.12) |  | 0.87(0.26, 1.49) |  | -0.49(-1.96, 0.98) | -0.29(-0.74, 0.16) | -0.23(-0.68, 0.22) |
| Low (Q1) | Ref. | Ref. |  | Ref. |  | Ref. | Ref. | Ref. |
| Medium (Q2) | 0.19(-0.01, 0.39) | 0.14(-0.09, 0.37) |  | -0.41(-2.49, 1.68) |  | -2.15(-7.15, 2.84) | -1.06(-2.58, 0.47) | -0.37(-1.90, 1.15) |
| High (Q3) | 0.39(0.17, 0.61) | 0.20(-0.06, 0.45) |  | 2.69(0.40, 4.99) |  | -2.63(-8.12, 2.85) | -1.42(-3.10, 0.25) | -0.83(-2.51, 0.84) |
| Early adolescence/per SD | 0.11(0.05, 0.18) | 0.10(0.03, 0.17) |  | 1.04(0.40, 1.68) |  | -1.10(-2.63, 0.43) | -0.49(-0.96, -0.03) | -0.26(-0.72, 0.21) |
| Low (Q1) | Ref. | Ref. |  | Ref. |  | Ref. | Ref. | Ref. |
| Medium (Q2) | 0.19(0.001, 0.39) | 0.29(0.07, 0.52) |  | 0.88(-1.15, 2.91) |  | -3.84(-8.30, 1.33) | -1.57(-3.04, -0.11) | -0.36(-1.83, 1.11) |
| High (Q3) | 0.36(0.14, 0.59) | 0.23(-0.03, 0.50) |  | 3.57(1.20, 5.93) |  | -2.78(-8.46, 2.90) | -1.43(-3.16, 0.30) | -0.60(-2.33, 1.14) |
| *Household wealth conditional gains between periods* | | | | | | | | |
| Gains from pregnancy to mid-childhood/per SD | 0.12(0.03, 0.21) | 0.06(-0.04, 0.16) |  | 0.97(0.05, 1.89) |  | 0.38(-1.82, 2.58) | -0.17(-0.85, 0.50) | 0.03(-0.64, 0.70) |
| Low (Q1) | Ref. | Ref. |  | Ref. |  | Ref. | Ref. | Ref. |
| Medium (Q2) | 0.03(-0.18, 0.23) | 0.11(-0.12, 0.35) |  | 0.80(-1.28, 2.89) |  | 0.96(-4.08, 6.00) | -0.62(-2.16, 0.92) | 0.19(-1.34, 1.73) |
| High (Q3) | 0.21(-0.004, 0.42) | 0.16(-0.08, 0.40) |  | 2.06(-0.11, 4.22) |  | -0.50(-5.68, 4.67) | -0.94(-2.52, 0.64) | -0.09(-1.66, 1.49) |
| <=0 | Ref. | Ref. |  | Ref. |  | Ref. | Ref. | Ref. |
| >0 | 0.24(0.07, 0.41) | 0.09(-0.11, 0.29) |  | 1.67(-0.10, 3.44) |  | 0.53(-3.71, 4.76) | -0.19(-1.49, 1.11) | 0.09(-1.20, 1.38) |
| Gains from mid-childhood to early adolescence/per SD | 0.06(-0.03, 0.14) | 0.11(0.01, 0.20) |  | 0.71(-0.17, 1.58) |  | -0.76(-2.84, 1.32) | -0.40(-1.04, 0.24) | -0.02(-0.66, 0.61) |
| Low (Q1) | Ref. | Ref. |  | Ref. |  | Ref. | Ref. | Ref. |
| Medium (Q2) | 0.14(-0.05, 0.34) | 0.40(0.18, 0.63) |  | 0.45(-1.59, 2.49) |  | -0.89(-5.75, 3.97) | -0.81(-2.29, 0.68) | 0.22(-1.27, 1.70) |
| High (Q3) | 0.20(-0.005, 0.41) | 0.35(0.11, 0.59) |  | 1.60(-0.56, 3.76) |  | -0.09(-5.23, 5.06) | -0.39(-1.96, 1.18) | 0.42(-1.15, 1.99) |
| <=0 | Ref. | Ref. |  | Ref. |  | Ref. | Ref. | Ref. |
| >0 | 0.07(-0.09, 0.24) | 0.22(0.03, 0.41) |  | 0.83(-0.90, 2.56) |  | -1.52(-5.67, 2.62) | -0.45(-1.71, 0.82) | -0.26(-1.53, 1.00) |
| Gains from pregnancy to early adolescence/per SD | 0.12(0.03, 0.21) | 0.13(0.03, 0.24) |  | 1.18(0.24, 2.13) |  | -0.45(-2.71, 1.81) | -0.46(-1.15, 0.23) | 0.02(-0.67, 0.71) |
| Low (Q1) | Ref. | Ref. |  | Ref. |  | Ref. | Ref. | Ref. |
| Medium (Q2) | 0.07(-0.12, 0.26) | 0.34(0.12, 0.56) |  | -0.51(-2.50, 1.49) |  | -2.15(-6.91, 2.62) | -1.10(-2.55, 0.36) | 0.11(-1.35, 1.56) |
| High (Q3) | 0.31(0.09, 0.52) | 0.35(0.10, 0.60) |  | 2.54(0.29, 4.78) |  | -2.11(-7.48, 3.26) | -1.38(-3.02, 0.26) | -0.34(-1.98, 1.30) |
| <=0 | Ref. | Ref. |  | Ref. |  | Ref. | Ref. | Ref. |
| >0 | 0.11(-0.06, 0.28) | 0.24(0.04, 0.43) |  | 0.62(-1.13, 2.36) |  | -1.88(-6.07, 2.32) | -0.91(-2.19, 0.37) | -0.50(-1.78, 0.78) |
| *Trajectories* | | | | | | | | |
| Upward versus Consistently low | 0.16(-0.13, 0.45) | 0.10(-0.24, 0.44) |  | 4.44(1.44, 7.44) |  | -0.89(-8.14, 6.35) | -1.18(-3.40, 1.03) | -0.23(-2.44, 1.98) |
| Downward versus Consistently low | 0.15(-0.08, 0.37) | -0.07(-0.33, 0.19) |  | 2.60(0.29, 4.91) |  | -4.15(-9.77, 1.46) | -1.52(-3.23, 0.20) | -1.28(-2.99, 0.43) |
| Consistently high versus Consistently low | 0.62(0.30, 0.94) | 0.28(-0.09, 0.65) |  | 4.55(1.19, 7.90) |  | -5.40(-13.71, 2.91) | -1.54(-4.08, 1.00) | -2.57(-5.10, -0.04) |
| Upward versus Consistently high | -0.46(-0.83, -0.09) | -0.17(-0.61, 0.26) |  | -0.11(-3.98, 3.77) |  | 4.50(-5.10, 14.11) | 0.36(-2.57, 3.30) | 2.34(-0.59, 5.27) |
| Downward versus Consistently high | -0.47(-0.79, -0.15) | -0.35(-0.72, 0.02) |  | -1.95(-5.29, 1.39) |  | 1.24(-7.19, 9.68) | 0.02(-2.55, 2.60) | 1.29(-1.28, 3.86) |
| Upward versus Downward | 0.01(-0.30, 0.33) | 0.17(-0.20, 0.54) |  | 1.84(-1.43, 5.11) |  | 3.26(-4.73, 11.25) | 0.34(-2.10, 2.78) | 1.05(-1.38, 3.49) |

Abbreviations: HAZ, height-for- age and sex z score; BAZ, body mass index-for- age and sex z score; FSIQ, full-scale intelligent quotient.

^a^Data are presented with adjusted mean differences and their 95% confidence intervals. The adjustments included parental education, occupation and age, maternal parity and mid-upper arm circumference, randomized regimens by duration, small-for-gestational age, and adolescent age.

Supplemental Table 10 Stratified analysis on associations between household wealth mobility and HAZ, BAZ, cognitive development, emotional and behavioral problems among adolescent females in a birth cohort in rural western China (*n*=480)

| Household wealth | *Z* scores of physical growth^a^ | |  | Cognitive development^a^ |  | Scores of emotional and behavioral problems^a^ | | |
| --- | --- | --- | --- | --- | --- | --- | --- | --- |
|  | HAZ | BAZ |  | FISQ |  | Total problem | Internalizing | Externalizing |
| *Household wealth index at single-time point* | | | | | | | | |
| Pregnancy/per SD | 0.08(-0.004, 0.16) | 0.04(-0.04, 0.13) |  | 0.84(-0.17, 1.84) |  | -0.32(-2.18, 1.54) | -0.04(-0.69, 0.61) | -0.02(-0.50, 0.47) |
| Low (Q1) | Ref. | Ref. |  | Ref. |  | Ref. | Ref. | Ref. |
| Medium (Q2) | -0.04(-0.28, 0.20) | 0.02(-0.24, 0.27) |  | 0.21(-2.75, 3.17) |  | 1.86(-3.55, 7.27) | 1.01(-0.88, 2.91) | 0.42(-0.99, 1.84) |
| High (Q3) | 0.23(-0.05, 0.50) | 0.14(-0.15, 0.44) |  | 2.94(-0.47, 6.35) |  | -1.45(-7.76, 4.86) | -0.28(-2.49, 1.93) | -0.37(-2.02, 1.28) |
| Mid-childhood/per SD | 0.09(0.02, 0.16) | -0.004(-0.08, 0.07) |  | 1.63(0.82, 2.44) |  | 0.63(-0.88, 2.16) | 0.10(-0.44, 0.63) | 0.17(-0.23, 0.57) |
| Low (Q1) | Ref. | Ref. |  | Ref. |  | Ref. | Ref. | Ref. |
| Medium (Q2) | 0.15(-0.08, 0.38) | 0.04(-0.20, 0.29) |  | 1.07(-1.76, 3.90) |  | 3.79(-1.46, 9.03) | 1.55(-0.28, 3.39) | 0.74(-0.63, 2.11) |
| High (Q3) | 0.41(0.16, 0.66) | -0.12(-0.38, 0.15) |  | 4.30(1.20, 7.41) |  | 2.30(-3.54, 8.15) | 0.58(-1.46, 2.63) | 0.47(-1.06, 2.00) |
| Early adolescence/per SD | 0.10(0.03, 0.17) | -0.01(-0.08, 0.07) |  | 2.08(1.23, 2.93) |  | 0.72(-0.89, 2.34) | 0.12(-0.45, 0.69) | 0.10(-0.32, 0.53) |
| Low (Q1) | Ref. | Ref. |  | Ref. |  | Ref. | Ref. | Ref. |
| Medium (Q2) | 0.29(0.06, 0.52) | 0.16(-0.09, 0.40) |  | 1.34(-1.39, 4.07) |  | -1.24(-6.44, 3.96) | -0.28(-2.10, 1.55) | 0.15(-1.21, 1.51) |
| High (Q3) | 0.30(0.04, 0.56) | 0.01(-0.27, 0.29) |  | 7.26(4.10, 10.42) |  | 1.09(-4.90, 7.09) | -0.30(-2.40, 1.80) | 0.04(-1.53, 1.61) |
| *Household wealth conditional gains between periods* | | | | | | | | |
| Gains from pregnancy to mid-childhood/per SD | 0.09(-0.003, 0.19) | -0.03(-0.13, 0.08) |  | 1.98(0.78, 3.18) |  | 1.08(-1.17, 3.33) | 0.16(-0.63, 0.95) | 0.25(-0.34, 0.84) |
| Low (Q1) | Ref. | Ref. |  | Ref. |  | Ref. | Ref. | Ref. |
| Medium (Q2) | 0.12(-0.11, 0.35) | -0.02(-0.27, 0.22) |  | 0.84(-1.95, 3.63) |  | -0.57(-5.82, 4.67) | 0.37(-1.47, 2.21) | -0.62(-1.99, 0.75) |
| High (Q3) | 0.25(0.01, 0.49) | -0.09(-0.34, 0.17) |  | 3.78(0.81, 6.74) |  | 2.58(-2.98, 8.14) | 0.67(-1.28, 2.62) | 0.51(-0.95, 1.96) |
| <=0 | Ref. | Ref. |  | Ref. |  | Ref. | Ref. | Ref. |
| >0 | 0.24(0.04, 0.44) | 0.003(-0.21, 0.22) |  | 2.01(-0.44, 4.46) |  | 3.18(-1.39, 7.75) | 0.72(-0.89, 2.32) | 0.96(-0.24, 2.15) |
| Gains from mid-childhood to early adolescence/per SD | 0.05(-0.04, 0.14) | -0.02(-0.12, 0.08) |  | 1.44(0.32, 2.56) |  | 0.56(-1.54, 2.67) | 0.10(-0.64, 0.84) | 0.02(-0.53, 0.57) |
| Low (Q1) | Ref. | Ref. |  | Ref. |  | Ref. | Ref. | Ref. |
| Medium (Q2) | 0.23(-0.01, 0.46) | 0.11(-0.14, 0.36) |  | 0.40(-2.46, 3.27) |  | 2.32(-3.05, 7.70) | 1.16(-0.72, 3.04) | -0.04(-1.45, 1.37) |
| High (Q3) | 0.17(-0.06, 0.40) | -0.04(-0.28, 0.20) |  | 3.84(1.03, 6.66) |  | 1.46(-3.81, 6.73) | -0.13(-1.97, 1.71) | 0.48(-0.90, 1.86) |
| <=0 | Ref. | Ref. |  | Ref. |  | Ref. | Ref. | Ref. |
| >0 | 0.14(-0.05, 0.34) | -0.01(-0.21, 0.19) |  | 2.59(0.24, 4.94) |  | 2.21(-2.18, 6.61) | 0.49(-1.05, 2.03) | 0.33(-0.82, 1.48) |
| Gains from pregnancy to early adolescence/per SD | 0.09(-0.01, 0.19) | -0.03(-0.14, 0.07) |  | 2.33(1.14, 3.51) |  | 1.09(-1.16, 3.33) | 0.18(-0.61, 0.96) | 0.14(-0.44, 0.73) |
| Low (Q1) | Ref. | Ref. |  | Ref. |  | Ref. | Ref. | Ref. |
| Medium (Q2) | 0.28(0.05, 0.51) | 0.02(-0.22, 0.26) |  | 2.06(-0.72, 4.85) |  | -0.04(-5.33, 5.24) | 0.11(-1.74, 1.96) | 0.26(-1.12, 1.64) |
| High (Q3) | 0.27(0.03, 0.50) | -0.09(-0.34, 0.16) |  | 5.66(2.75, 8.57) |  | 2.06(-3.48, 7.60) | 0.11(-1.83, 2.06) | 0.13(-1.32, 1.58) |
| <=0 | Ref. | Ref. |  | Ref. |  | Ref. | Ref. | Ref. |
| >0 | 0.21(0.02, 0.41) | -0.05(-0.25, 0.16) |  | 3.83(1.47, 6.20) |  | 0.25(-4.22, 4.71) | -0.36(-1.92, 1.21) | -0.21(-1.37, 0.96) |
| *Trajectories* | | | | | | | | |
| Upward versus Consistently low | 0.38(0.05, 0.71) | -0.11(-0.46, 0.24) |  | 5.70(1.72, 9.68) |  | 2.33(-5.39, 10.05) | 0.45(-2.25, 3.16) | 0.62(-1.40, 2.64) |
| Downward versus Consistently low | 0.26(-0.01, 0.54) | 0.05(-0.24, 0.34) |  | 2.83(-0.43, 6.09) |  | 2.35(-3.93, 8.62) | 0.30(-1.90, 2.49) | 0.64(-1.00, 2.28) |
| Consistently high versus Consistently low | 0.31(-0.05, 0.68) | -0.13(-0.52, 0.27) |  | 9.68(5.22, 14.14) |  | -0.32(-8.77, 8.14) | -0.46(-3.42, 2.51) | 0.06(-2.15, 2.27) |
| Upward versus Consistently high | 0.06(-0.36, 0.49) | 0.01(-0.44, 0.46) |  | -3.98(-9.12, 1.16) |  | 2.64(-7.47, 12.76) | 0.91(-2.64, 4.46) | 0.56(-2.08, 3.21) |
| Downward versus Consistently high | -0.05(-0.40, 0.30) | 0.17(-0.20, 0.55) |  | -6.85(-11.14, -2.56) |  | 2.67(-5.69, 11.02) | 0.75(-2.17, 3.68) | 0.58(-1.61, 2.76) |
| Upward versus Downward | 0.11(-0.25, 0.48) | -0.16(-0.55, 0.23) |  | 2.87(-1.50, 7.24) |  | -0.02(-8.64, 8.60) | 0.16(-2.86, 3.18) | -0.02(-2.27, 2.24) |

Abbreviations: HAZ, height-for- age and sex z score; BAZ, body mass index-for- age and sex z score; FSIQ, full-scale intelligent quotient.

^a^Data are presented with adjusted mean differences and their 95% confidence intervals. The adjustments included parental education, occupation and age, maternal parity and mid-upper arm circumference, randomized regimens by duration, small-for-gestational age, and adolescent age.

Supplemental Table 11 Associations between household wealth mobility among periods and adolescent HAZ, BAZ, cognitive development, and emotional and behavioral problems from a birth cohort in rural western China after performing inverse probability weighting (*n*=1188)

| Household wealth | *Z* scores of physical growth^a^ | |  | Cognitive development^a^ |  | Scores of emotional and behavioral problems^a^ | | |
| --- | --- | --- | --- | --- | --- | --- | --- | --- |
|  | HAZ | BAZ |  | FISQ |  | Total problem | Internalizing | Externalizing |
| *Household wealth index at single-time point* | | | | | | | | |
| Pregnancy/per SD | 0.09(0.03, 0.14) | 0.03(-0.04, 0.09) |  | 0.67(0.08, 1.25) |  | -1.72(-3.06, -0.38) | -0.36(-0.78, 0.07) | -0.52(-0.91, -0.13) |
| Low (Q1) | Ref. | Ref. |  | Ref. |  | Ref. | Ref. | Ref. |
| Medium (Q2) | 0.04(-0.11, 0.19) | -0.04(-0.21, 0.13) |  | 0.11(-1.56, 1.77) |  | -2.95(-6.72, 0.82) | -0.45(-1.69, 0.78) | -0.61(-1.72, 0.50) |
| High (Q3) | 0.24(0.06, 0.42) | 0.12(-0.09, 0.33) |  | 2.33(0.35, 4.30) |  | -5.75(-10.27, -1.23) | -1.41(-2.85, 0.02) | -1.92(-3.26, -0.59) |
| Mid-childhood per SD | 0.10(0.06, 0.14) | 0.02(-0.03, 0.07) |  | 1.15(0.64, 1.66) |  | 0.07(-1.07, 1.20) | -0.09(-0.46, 0.27) | -0.06(-0.37, 0.26) |
| Low (Q1) | Ref. | Ref. |  | Ref. |  | Ref. | Ref. | Ref. |
| Medium (Q2) | 0.15(0.001, 0.29) | 0.08(-0.09, 0.25) |  | 0.17(-1.53, 1.87) |  | 0.55(-3.16, 4.25) | 0.09(-1.14, 1.31) | 0.10(-0.96, 1.16) |
| High (Q3) | 0.37(0.20, 0.53) | 0.03(-0.16, 0.22) |  | 3.29(1.50, 5.09) |  | -0.30(-4.25, 3.66) | -0.48(-1.76, 0.81) | -0.31(-1.45, 0.83) |
| Early adolescence/per SD | 0.11(0.06, 0.16) | 0.05(-0.001, 0.11) |  | 1.48(0.94, 2.02) |  | -0.49(-1.60, 0.63) | -0.29(-0.66, 0.08) | -0.16(-0.48, 0.15) |
| Low (Q1) | Ref. | Ref. |  | Ref. |  | Ref. | Ref. | Ref. |
| Medium (Q2) | 0.22(0.08, 0.36) | 0.25(0.08, 0.41) |  | 1.14(-0.49, 2.77) |  | -2.01(-5.67, 1.66) | -1.04(-2.24, 0.16) | 0.05(-1.00, 1.11) |
| High (Q3) | 0.35(0.18, 0.51) | 0.16(-0.04, 0.35) |  | 5.28(3.25, 7.31) |  | -1.77(-5.85, 2.32) | -1.23(-2.58, 0.12) | -0.53(-1.66, 0.60) |
| *Household wealth conditional gains between periods* | | | | | | | | |
| Gains from pregnancy to mid-childhood/per SD | 0.11(0.04, 0.17) | 0.02(-0.06, 0.10) |  | 1.38(0.60, 2.15) |  | 0.89(-0.86, 2.63) | 0.02(-0.53, 0.58) | 0.16(-0.34, 0.65) |
| Low (Q1) | Ref. | Ref. |  | Ref. |  | Ref. | Ref. | Ref. |
| Medium (Q2) | 0.05(-0.10, 0.20) | 0.05(-0.12, 0.22) |  | 0.93(-0.79, 2.65) |  | 0.58(-3.06, 4.22) | -0.19(-1.41, 1.02) | -0.09(-1.10, 0.92) |
| High (Q3) | 0.20(0.04, 0.36) | 0.04(-0.15, 0.22) |  | 2.91(1.18, 4.63) |  | 1.32(-2.49, 5.12) | -0.18(-1.37, 1.02) | 0.25(-0.87, 1.38) |
| <=0 | Ref. | Ref. |  | Ref. |  | Ref. | Ref. | Ref. |
| >0 | 0.23(0.10, 0.36) | 0.03(-0.12, 0.18) |  | 1.85(0.40, 3.31) |  | 1.80(-1.36, 4.97) | 0.22(-0.80, 1.23) | 0.49(-0.43, 1.41) |
| Gains from mid-childhood to early adolescence/per SD | 0.06(-0.003, 0.12) | 0.05(-0.02, 0.12) |  | 1.08(0.36, 1.81) |  | -0.40(-1.88, 1.07) | -0.28(-0.74, 0.19) | -0.09(-0.49, 0.32) |
| Low (Q1) | Ref. | Ref. |  | Ref. |  | Ref. | Ref. | Ref. |
| Medium (Q2) | 0.20(0.05, 0.35) | 0.32(0.16, 0.49) |  | 0.90(-0.81, 2.61) |  | 0.56(-3.06, 4.18) | -0.01(-1.18, 1.17) | 0.15(-0.88, 1.19) |
| High (Q3) | 0.21(0.07, 0.36) | 0.20(0.02, 0.37) |  | 2.69(0.82, 4.56) |  | -0.06(-3.83, 3.72) | -0.52(-1.73, 0.68) | 0.25(-0.84, 1.33) |
| <=0 | Ref. | Ref. |  | Ref. |  | Ref. | Ref. | Ref. |
| >0 | 0.12(-0.002, 0.25) | 0.14(-0.001, 0.28) |  | 1.75(0.29, 3.21) |  | -0.87(-3.89, 2.14) | -0.34(-1.32, 0.63) | -0.28(-1.14, 0.59) |
| Gains from pregnancy to early adolescence/per SD | 0.11(0.04, 0.18) | 0.06(-0.02, 0.14) |  | 1.74(0.97, 2.52) |  | 0.13(-1.55, 1.81) | -0.24(-0.78, 0.30) | 0.01(-0.46, 0.49) |
| Low (Q1) | Ref. | Ref. |  | Ref. |  | Ref. | Ref. | Ref. |
| Medium (Q2) | 0.15(0.01, 0.30) | 0.25(0.09, 0.42) |  | 0.61(-1.11, 2.32) |  | -1.28(-4.92, 2.36) | -0.71(-1.89, 0.46) | 0.19(-0.86, 1.25) |
| High (Q3) | 0.30(0.14, 0.46) | 0.17(-0.02, 0.36) |  | 3.98(2.08, 5.87) |  | -0.79(-4.67, 3.09) | -0.96(-2.22, 0.29) | -0.35(-1.44, 0.74) |
| <=0 | Ref. | Ref. |  | Ref. |  | Ref. | Ref. | Ref. |
| >0 | 0.17(0.04, 0.29) | 0.12(-0.02, 0.27) |  | 2.03(0.54, 3.52) |  | -1.19(-4.29, 1.91) | -0.80(-1.79, 0.20) | -0.50(-1.38, 0.38) |
| *Trajectories* | | | | | | | | |
| Upward versus Consistently low | 0.24(0.01, 0.46) | -0.06(-0.32, 0.20) |  | 5.14(2.95, 7.32) |  | 0.51(-4.87, 5.89) | -0.52(-2.26, 1.21) | 0.14(-1.57, 1.86) |
| Downward versus Consistently low | 0.19(0.03, 0.35) | -0.04(-0.24, 0.15) |  | 2.40(0.55, 4.25) |  | -2.04(-6.31, 2.23) | -0.89(-2.31, 0.53) | -0.74(-1.93, 0.44) |
| Consistently high versus Consistently low | 0.49(0.24, 0.73) | 0.08(-0.20, 0.37) |  | 6.63(3.83, 9.42) |  | -3.88(-9.92, 2.15) | -1.15(-3.10, 0.80) | -1.62(-3.22, -0.02) |
| Upward versus Consistently high | -0.25(-0.55, 0.05) | -0.15(-0.48, 0.19) |  | -1.49(-4.58, 1.60) |  | 4.39(-2.86, 11.65) | 0.63(-1.65, 2.90) | 1.76(-0.43, 3.96) |
| Downward versus Consistently high | -0.30(-0.54, -0.05) | -0.13(-0.42, 0.16) |  | -4.23(-7.15, -1.30) |  | 1.84(-3.92, 7.61) | 0.26(-1.57, 2.08) | 0.88(-0.65, 2.41) |
| Upward versus Downward | 0.05(-0.20, 0.29) | -0.02(-0.31, 0.28) |  | 2.74(0.23, 5.24) |  | 2.55(-3.63, 8.73) | 0.37(-1.59, 2.32) | 0.88(-1.07, 2.84) |

Abbreviations: HAZ, height-for- age and sex z score; BAZ, body mass index-for- age and sex *z* score; FSIQ, full-scale intelligent quotient.

^a^Data are presented with adjusted mean differences and their 95% confidence intervals. The adjustments included parental education, occupation and age, maternal parity and mid-upper arm circumference, randomized regimens by duration, small-for-gestational age, and adolescent sex and age.

Supplemental Table 12 Associations between household wealth mobility and adolescent HAZ, BAZ, cognitive development and emotional and behavioral problems among the lowest 80% household wealth at baseline from a birth cohort in rural western China (*n*=951)

| Household wealth | *Z* scores of physical growth^a^ | |  | Cognitive development^a^ |  | Emotional and behavioral problems^a^ | | |
| --- | --- | --- | --- | --- | --- | --- | --- | --- |
|  | HAZ | BAZ |  | FISQ |  | Total problem | Internalizing | Externalizing |
| *Household wealth index at single-time point* | | | | | | | | |
| Pregnancy/per SD | 0.08(0.01, 0.15) | 0.01(-0.07, 0.09) |  | 0.69(-0.11, 1.48) |  | -1.69(-3.43, 0.06) | -0.37(-0.93, 0.19) | -0.54(-1.06, -0.03) |
| Low (Q1) | Ref. | Ref. |  | Ref. |  | Ref. | Ref. | Ref. |
| Medium (Q2) | 0.09(-0.06, 0.24) | 0.01(-0.16, 0.17) |  | 0.06(-1.59, 1.72) |  | -3.39(-7.03, 0.25) | -0.52(-1.69, 0.65) | -0.65(-1.73, 0.42) |
| High (Q3) | 0.17(-0.07, 0.40) | 0.14(-0.12, 0.40) |  | 2.88(0.29, 5.47) |  | -6.68(-12.61, -1.12) | -1.92(-3.76, -0.07) | -2.29(-3.99, -0.60) |
| Mid-childhood/per SD | 0.08(0.03, 0.13) | 0.01(-0.04, 0.07) |  | 1.43(0.87, 1.98) |  | -0.54(-1.77, 0.70) | -0.35(-0.74, 0.05) | -0.12(-0.48, 0.25) |
| Low (Q1) | Ref. | Ref. |  | Ref. |  | Ref. | Ref. | Ref. |
| Medium (Q2) | 0.18(0.02, 0.33) | 0.11(-0.06, 0.29) |  | 0.89(-0.85, 2.63) |  | -0.95(-4.83, 2.92) | -0.37(-1.61, 0.88) | -0.18(-1.33, 0.97) |
| High (Q3) | 0.35(0.17, 0.53) | 0.09(-0.11, 0.30) |  | 3.77(1.75, 5.79) |  | -1.87(-6.37, 2.62) | -1.22(-2.66, 0.22) | -0.37(-1.70, 0.95) |
| Early adolescence/per SD | 0.10(0.05, 0.16) | 0.05(-0.01, 0.11) |  | 1.50(0.92, 2.08) |  | -1.14(-2.44, 0.16) | -0.45(-0.87, 0.03) | -0.28(-0.66, 0.10) |
| Low (Q1) | Ref. | Ref. |  | Ref. |  | Ref. | Ref. | Ref. |
| Medium (Q2) | 0.22(0.07, 0.37) | 0.23(0.06, 0.40) |  | 1.27(-0.43, 2.96) |  | -3.12(-6.90, 0.66) | -1.22(-2.43, -0.01) | -0.29(-1.41, 0.83) |
| High (Q3) | 0.32(0.13, 0.51) | 0.10(-0.12, 0.31) |  | 4.78(2.64, 6.92) |  | -3.16(-7.96, 1.64) | -1.41(-2.95, 0.13) | -0.93(-2.35, 0.49) |
| *Household wealth conditional gains between periods* | | | | | | | | |
| Gains from pregnancy to mid-childhood/per SD | 0.07(-0.001, 0.15) | 0.02(-0.06, 0.10) |  | 1.74(0.92, 2.57) |  | -0.10(-1.93, 1.72) | -0.34(-0.93, 0.24) | 0.04(-0.50, 0.58) |
| Low (Q1) | Ref. | Ref. |  | Ref. |  | Ref. | Ref. | Ref. |
| Medium (Q2) | 0.03(-0.13, 0.19) | 0.10(-0.08, 0.29) |  | 0.98(-0.83, 2.79) |  | -0.66(-4.69, 3.37) | -0.54(-1.84, 0.75) | -0.32(-1.51, 0.88) |
| High (Q3) | 0.16(-0.01, 0.34) | 0.06(-0.13, 0.26) |  | 2.82(0.87, 4.78) |  | -0.80(-5.12, 3.53) | -1.02(-2.41, 0.37) | 0.08(-1.19, 1.36) |
| <=0 | Ref. | Ref. |  | Ref. |  | Ref. | Ref. | Ref. |
| >0 | 0.21(0.06, 0.35) | 0.05(-0.11, 0.21) |  | 2.10(0.51, 3.69) |  | 0.42(-3.10, 3.94) | -0.36(-1.49, 0.77) | 0.35(-0.69, 1.39) |
| Gains from mid-childhood to early adolescence/per SD | 0.09(0.02, 0.16) | 0.06(-0.02, 0.14) |  | 0.93(0.14, 1.72) |  | -1.00(-2.74, 0.74) | -0.31(-0.87, 0.24) | -0.24(-0.76, 0.27) |
| Low (Q1) | Ref. | Ref. |  | Ref. |  | Ref. | Ref. | Ref. |
| Medium (Q2) | 0.18(0.02, 0.34) | 0.31(0.13, 0.49) |  | 0.71(-1.12, 2.54) |  | -0.21(-4.24, 3.81) | -0.09(-1.38, 1.21) | -0.14(-1.33, 1.05) |
| High (Q3) | 0.19(0.02, 0.36) | 0.18(-0.01, 0.37) |  | 1.81(-0.12, 3.73) |  | -1.49(-5.71, 2.73) | -0.61(-1.97, 0.75) | -0.14(-1.39, 1.11) |
| <=0 | Ref. | Ref. |  | Ref. |  | Ref. | Ref. | Ref. |
| >0 | 0.09(-0.05, 0.23) | 0.11(-0.04, 0.27) |  | 0.90(-0.64, 2.44) |  | -1.76(-5.16, 1.64) | -0.35(-1.44, 0.75) | -0.49(-1.50, 0.52) |
| Gains from pregnancy to early adolescence/per SD | 0.12(0.05, 0.20) | 0.07(-0.02, 0.15) |  | 1.86(1.00, 2.71) |  | -0.99(-2.89, 0.91) | -0.49(-1.10, 0.12) | -0.20(-0.77, 0.36) |
| Low (Q1) | Ref. | Ref. |  | Ref. |  | Ref. | Ref. | Ref. |
| Medium (Q2) | 0.15(-0.01, 0.30) | 0.28(0.10, 0.45) |  | 0.59(-1.17, 2.34) |  | -1.37(-5.28, 2.54) | -0.53(-1.78, 0.73) | 0.09(-1.07, 1.25) |
| High (Q3) | 0.29(0.11, 0.47) | 0.17(-0.04, 0.37) |  | 3.76(1.74, 5.78) |  | -3.32(-7.80, 1.15) | -1.66(-3.10, -0.23) | -0.70(-2.02, 0.63) |
| <=0 | Ref. | Ref. |  | Ref. |  | Ref. | Ref. | Ref. |
| >0 | 0.16(0.02, 0.30) | 0.12(-0.03, 0.28) |  | 1.63(0.07, 3.19) |  | -2.82(-6.28, 0.63) | -1.08(-2.20, 0.03) | -0.82(-1.84, 0.21) |
| *Trajectories* | | | | | | | | |
| Upward versus Consistently low | 0.29(0.08, 0.51) | 0.04(-0.21, 0.28) |  | 4.91(2.51, 7.31) |  | -1.13(-6.58, 4.31) | -0.90(-2.65, 0.85) | -0.24(-1.85, 1.37) |
| Downward versus Consistently low | 0.19(-0.02, 0.39) | -0.11(-0.34, 0.12) |  | 2.64(0.36, 4.92) |  | -2.54(-7.74, 2.66) | -1.07(-2.74, 0.60) | -0.62(-2.16, 0.92) |
| Consistently high versus Consistently low | 0.27(-0.15, 0.68) | 0.18(-0.29, 0.64) |  | 9.28(4.70, 13.85) |  | -7.45(-17.75, 2.85) | -2.32(-5.63, 0.99) | -2.50(-5.54, 0.55) |
| Upward versus Consistently high | 0.03(-0.41, 0.46) | -0.14(-0.63, 0.35) |  | -4.37(-9.18, 0.44) |  | 6.32(-4.60, 17.23) | 1.42(-2.09, 4.93) | 2.26(-0.97, 5.49) |
| Downward versus Consistently high | -0.08(-0.52, 0.35) | -0.29(-0.77, 0.20) |  | -6.63(-11.43, -1.84) |  | 4.91(-5.93, 15.76) | 1.25(-2.24, 4.73) | 1.88(-1.33, 5.09) |
| Upward versus Downward | 0.11(-0.16, 0.37) | 0.14(-0.15, 0.44) |  | 2.27(-0.64, 5.17) |  | 1.41(-5.25, 8.07) | 0.17(-1.97, 2.31) | 0.38(-1.60, 2.35) |

Abbreviations: HAZ, height-for- age and sex z score; BAZ, body mass index-for- age and sex *z* score; FSIQ, full-scale intelligent quotient.

^a^Data are presented with adjusted mean differences and their 95% confidence intervals. The adjustments included parental education, occupation and age, maternal parity and mid-upper arm circumference, randomized regimens by durations, small-for-gestational age, and adolescent sex and age.

Supplemental Table 13 Associations between household wealth mobility and adolescent HAZ, BAZ, cognitive development and emotional and behavioral problems among the highest 80% household wealth at baseline from a birth cohort in rural western China (*n*=949)

| Household wealth | *Z* scores of physical growth^a^ | |  | Cognitive development^a^ |  | Emotional and behavioral problems^a^ | | |
| --- | --- | --- | --- | --- | --- | --- | --- | --- |
|  | HAZ | BAZ |  | FISQ |  | Total problem | Internalizing | Externalizing |
| *Household wealth index at single-time point* | | | | | | | | |
| Pregnancy/per SD | 0.13(0.05, 0.20) | 0.08(-0.003, 0.17) |  | 0.89(0.03, 1.75) |  | -1.88(-3.73, -0.02) | -0.47(-1.07, 0.13) | -0.48(-1.02, 0.05) |
| Low (Q1) | Ref. | Ref. |  | Ref. |  | Ref. | Ref. | Ref. |
| Medium (Q2) | 0.09(-0.10, 0.29) | 0.02(-0.19, 0.24) |  | -0.10(-2.29, 2.09) |  | -3.06(-7.65, 1.52) | -0.66(-2.13, 0.82) | -0.39(-1.71, 0.92) |
| High (Q3) | 0.27(0.05, 0.49) | 0.19(-0.06, 0.44) |  | 2.21(-0.27, 4.69) |  | -5.28(-10.48, -0.09) | -1.49(-3.16, 0.19) | -1.57(-3.06, -0.08) |
| Mid-childhood/per SD | 0.12(0.07, 0.17) | 0.03(-0.03, 0.08) |  | 1.28(0.74, 1.83) |  | 0.34(-0.83, 1.52) | 0.02(-0.36, 0.40) | -0.03(-0.36, 0.31) |
| Low (Q1) | Ref. | Ref. |  | Ref. |  | Ref. | Ref. | Ref. |
| Medium (Q2) | 0.14(-0.03, 0.32) | 0.03(-0.17, 0.23) |  | 0.70(-1.27, 2.66) |  | 0.45(-3.76, 4.65) | 0.05(-1.30, 1.41) | -0.11(-1.32, 1.10) |
| High (Q3) | 0.41(0.23, 0.60) | 0.04(-0.17, 0.25) |  | 3.82(1.74, 5.90) |  | 0.71(-3.77, 5.18) | -0.21(-1.65, 1.24) | -0.16(-1.44, 1.13) |
| Early adolescence/per SD | 0.11(0.06, 0.16) | 0.05(-0.002, 0.11) |  | 1.47(0.90, 2.03) |  | 0.01(-1.21, 1.23) | -0.12(-0.51, 0.27) | -0.06(-0.41, 0.29) |
| Low (Q1) | Ref. | Ref. |  | Ref. |  | Ref. | Ref. | Ref. |
| Medium (Q2) | 0.25(0.08, 0.42) | 0.30(0.11, 0.49) |  | 1.40(-0.51, 3.30) |  | -0.41(-4.51, 3.69) | -0.27(-1.60, 1.05) | 0.32(-0.85, 1.50) |
| High (Q3) | 0.36(0.18, 0.55) | 0.17(-0.04, 0.38) |  | 5.21(3.12, 7.31) |  | 0.44(-4.07, 4.96) | -0.55(-2.00, 0.90) | -0.02(-1.31, 1.27) |
| *Household wealth conditional gains between periods* | | | | | | | | |
| Gains from pregnancy to mid-childhood/per SD | 0.14(0.06, 0.21) | 0.02(-0.07, 0.10) |  | 1.69(0.86, 2.51) |  | 1.05(-0.72, 2.83) | 0.16(-0.41, 0.74) | 0.10(-0.41, 0.60) |
| Low (Q1) | Ref. | Ref. |  | Ref. |  | Ref. | Ref. | Ref. |
| Medium (Q2) | 0.10(-0.07, 0.27) | 0.04(-0.14, 0.23) |  | 1.31(-0.56, 3.18) |  | -0.49(-4.53, 3.54) | -0.39(-1.69, 0.91) | -0.52(-1.68, 0.63) |
| High (Q3) | 0.30(0.12, 0.48) | 0.03(-0.17, 0.23) |  | 3.80(1.79, 5.81) |  | 1.56(-2.78, 5.90) | 0.08(-1.32, 1.48) | 0.11(-1.13, 1.36) |
| <=0 | Ref. | Ref. |  | Ref. |  | Ref. | Ref. | Ref. |
| >0 | 0.30(0.15, 0.45) | 0.02(-0.15, 0.18) |  | 2.69(1.01, 4.36) |  | 2.18(-1.43, 5.80) | 0.51(-0.65, 1.68) | 0.32(-0.71, 1.36) |
| Gains from mid-childhood to early adolescence/per SD | 0.05(-0.02, 0.12) | 0.05(-0.03, 0.12) |  | 1.03(0.27, 1.80) |  | -0.03(-1.68, 1.61) | -0.14(-0.67, 0.39) | -0.01(-0.48, 0.47) |
| Low (Q1) | Ref. | Ref. |  | Ref. |  | Ref. | Ref. | Ref. |
| Medium (Q2) | 0.18(0.01, 0.35) | 0.25(0.06, 0.45) |  | 0.90(-1.02, 2.83) |  | 1.32(-2.79, 5.43) | 0.20(-1.12, 1.53) | 0.19(-0.99, 1.37) |
| High (Q3) | 0.16(-0.01, 0.33) | 0.16(-0.03, 0.35) |  | 2.83(0.91, 4.76) |  | 0.87(-3.23, 4.98) | -0.14(-1.47, 1.18) | 0.40(-0.78, 1.57) |
| <=0 | Ref. | Ref. |  | Ref. |  | Ref. | Ref. | Ref. |
| >0 | 0.11(-0.03, 0.26) | 0.13(-0.03, 0.29) |  | 1.95(0.36, 3.54) |  | 0.16(-3.26, 3.58) | 0.02(-1.08, 1.12) | -0.07(-1.05, 0.91) |
| Gains from pregnancy to early adolescence/per SD | 0.12(0.04, 0.19) | 0.05(-0.03, 0.14) |  | 1.84(1.01, 2.67) |  | 0.55(-1.23, 2.33) | -0.04(-0.61, 0.53) | 0.05(-0.46, 0.56) |
| Low (Q1) | Ref. | Ref. |  | Ref. |  | Ref. | Ref. | Ref. |
| Medium (Q2) | 0.18(0.01, 0.34) | 0.24(0.06, 0.42) |  | 0.80(-1.03, 2.63) |  | -1.13(-5.08, 2.82) | -0.65(-1.93, 0.62) | 0.15(-0.99, 1.28) |
| High (Q3) | 0.31(0.13, 0.49) | 0.16(-0.05, 0.36) |  | 4.45(2.40, 6.50) |  | 0.63(-3.81, 5.06) | -0.16(-1.59, 1.26) | -0.20(-1.47, 1.07) |
| <=0 | Ref. | Ref. |  | Ref. |  | Ref. | Ref. | Ref. |
| >0 | 0.15(0.01, 0.30) | 0.11(-0.05, 0.28) |  | 2.49(0.86, 4.11) |  | -0.41(-3.93, 3.10) | -0.32(-1.46, 0.81) | -0.52(-1.53, 0.49) |
| *Trajectories* | | | | | | | | |
| Upward versus Consistently low | 0.32(0.08, 0.57) | -0.01(-0.29, 0.26) |  | 4.63(1.91, 7.34) |  | 3.56(-2.38, 9.50) | 0.31(-1.61, 2.23) | 1.04(-0.66, 2.74) |
| Downward versus Consistently low | 0.20(0.02, 0.38) | -0.004(-0.21, 0.20) |  | 2.50(0.49, 4.51) |  | -0.27(-4.63, 4.08) | -0.56(-1.96, 0.85) | -0.11(-1.35, 1.14) |
| Consistently high versus Consistently low | 0.52(0.27, 0.77) | 0.13(-0.15, 0.41) |  | 6.46(3.65, 9.26) |  | -2.63(-8.75, 3.48) | -0.82(-2.79, 1.15) | -1.24(-3.00, 0.51) |
| Upward versus Consistently high | -0.20(-0.50, 0.10) | -0.14(-0.48, 0.19) |  | -1.83(-5.16, 1.50) |  | 6.19(-1.22, 13.59) | 1.13(-1.26, 3.52) | 2.28(0.16, 4.40) |
| Downward versus Consistently high | -0.32(-0.56, -0.08) | -0.13(-0.41, 0.14) |  | -3.96(-6.65, -1.26) |  | 2.36(-3.64, 8.37) | 0.26(-1.67, 2.20) | 1.14(-0.58, 2.86) |
| Upward versus Downward | 0.12(-0.14, 0.38) | -0.01(-0.30, 0.28) |  | 2.13(-0.74, 4.99) |  | 3.83(-2.50, 10.15) | 0.87(-1.17, 2.91) | 1.14(-0.67, 2.96) |

Abbreviations: HAZ, height-for- age and sex z score; BAZ, body mass index-for- age and sex *z* score; FSIQ, full-scale intelligent quotient.

^a^Data are presented with adjusted mean differences and their 95% confidence intervals. The adjustments included parental education, occupation and age, maternal parity and mid-upper arm circumference, randomized regimens by durations, small-for-gestational age, and adolescent sex and age.

Supplemental Table 14 E-values for associations between household wealth mobility and adolescent HAZ, BAZ, cognitive development and emotional and behavioral problems from a birth cohort in rural western China (*n*=1188)

| Household wealth | *Z* scores of physical growth^a^ | |  | Cognitive development^a^ |  | Emotional and behavioral problems^a^ | | |
| --- | --- | --- | --- | --- | --- | --- | --- | --- |
|  | HAZ | BAZ |  | FISQ |  | Total problem | Internalizing | Externalizing |
| *Household wealth index at single-time point* | | | | | | | | |
| Pregnancy/per SD | 1.31 | 1.16 |  | 2.53 |  | 4.84 | 1.75 | 2.05 |
| Low (Q1) | Ref. | Ref. |  | Ref. |  | Ref. | Ref. | Ref. |
| Medium (Q2) | 1.28 | 1.14 |  | 1.03 |  | 1.47 | 1.29 | 1.36 |
| High (Q3) | 1.75 | 1.43 |  | 1.69 |  | 1.70 | 1.59 | 1.78 |
| Mid-childhood/per SD | 1.31 | 1.14 |  | 3.33 |  | 1.08 | 1.33 | 1.23 |
| Low (Q1) | Ref. | Ref. |  | Ref. |  | Ref. | Ref. | Ref. |
| Medium (Q2) | 1.55 | 1.38 |  | 1.11 |  | 1.15 | 1.10 | 1.14 |
| High (Q3) | 2.10 | 1.30 |  | 1.89 |  | 1.12 | 1.31 | 1.20 |
| Early adolescence/per SD | 1.33 | 1.22 |  | 4.25 |  | 1.99 | 1.64 | 1.40 |
| Low (Q1) | Ref. | Ref. |  | Ref. |  | Ref. | Ref. | Ref. |
| Medium (Q2) | 1.70 | 1.71 |  | 1.40 |  | 1.37 | 1.48 | 1.08 |
| High (Q3) | 2.00 | 1.48 |  | 2.32 |  | 1.32 | 1.52 | 1.31 |
| *Household wealth conditional gains between periods* | | | | | | | | |
| Gains from pregnancy to mid-childhood/per SD | 1.45 | 1.19 |  | 6.79 |  | 3.22 | 1.14 | 1.51 |
| Low (Q1) | Ref. | Ref. |  | Ref. |  | Ref. | Ref. | Ref. |
| Medium (Q2) | 1.22 | 1.30 |  | 1.33 |  | 1.15 | 1.16 | 1.12 |
| High (Q3) | 1.67 | 1.27 |  | 1.77 |  | 1.23 | 1.19 | 1.20 |
| <=0 | Ref. | Ref. |  | Ref. |  | Ref. | Ref. | Ref. |
| >0 | 1.75 | 1.24 |  | 1.59 |  | 1.31 | 1.17 | 1.30 |
| Gains from mid-childhood to early adolescence/per SD | 1.29 | 1.27 |  | 4.73 |  | 2.16 | 1.83 | 1.29 |
| Low (Q1) | Ref. | Ref. |  | Ref. |  | Ref. | Ref. | Ref. |
| Medium (Q2) | 1.57 | 1.80 |  | 1.27 |  | 1.13 | 1.05 | 1.13 |
| High (Q3) | 1.62 | 1.59 |  | 1.72 |  | 1.02 | 1.29 | 1.23 |
| <=0 | Ref. | Ref. |  | Ref. |  | Ref. | Ref. | Ref. |
| >0 | 1.42 | 1.43 |  | 1.52 |  | 1.16 | 1.19 | 1.17 |
| Gains from pregnancy to early adolescence/per SD | 1.46 | 1.32 |  | 9.92 |  | 1.30 | 1.82 | 1.19 |
| Low (Q1) | Ref. | Ref. |  | Ref. |  | Ref. | Ref. | Ref. |
| Medium (Q2) | 1.50 | 1.69 |  | 1.26 |  | 1.23 | 1.33 | 1.20 |
| High (Q3) | 1.87 | 1.55 |  | 2.02 |  | 1.19 | 1.45 | 1.22 |
| <=0 | Ref. | Ref. |  | Ref. |  | Ref. | Ref. | Ref. |
| >0 | 1.52 | 1.43 |  | 1.58 |  | 1.26 | 1.40 | 1.31 |
| *Trajectories* | | | | | | | | |
| Upward versus Consistently low | 1.77 | 1.10 |  | 2.26 |  | 1.09 | 1.33 | 1.11 |
| Downward versus Consistently low | 1.62 | 1.18 |  | 1.73 |  | 1.29 | 1.40 | 1.31 |
| Consistently high versus Consistently low | 2.40 | 1.38 |  | 2.70 |  | 1.54 | 1.51 | 1.70 |
| Upward versus Consistently high | 1.75 | 1.40 |  | 1.52 |  | 1.56 | 1.31 | 1.73 |
| Downward versus Consistently high | 1.92 | 1.45 |  | 2.05 |  | 1.38 | 1.23 | 1.53 |
| Upward versus Downward | 1.34 | 1.10 |  | 1.65 |  | 1.24 | 1.07 | 1.28 |

Abbreviations: HAZ, height-for- age and sex z score; BAZ, body mass index-for- age and sex *z* score; FSIQ, full-scale intelligent quotient.

E-values are calculated based on the results displayed in Table 2-4.


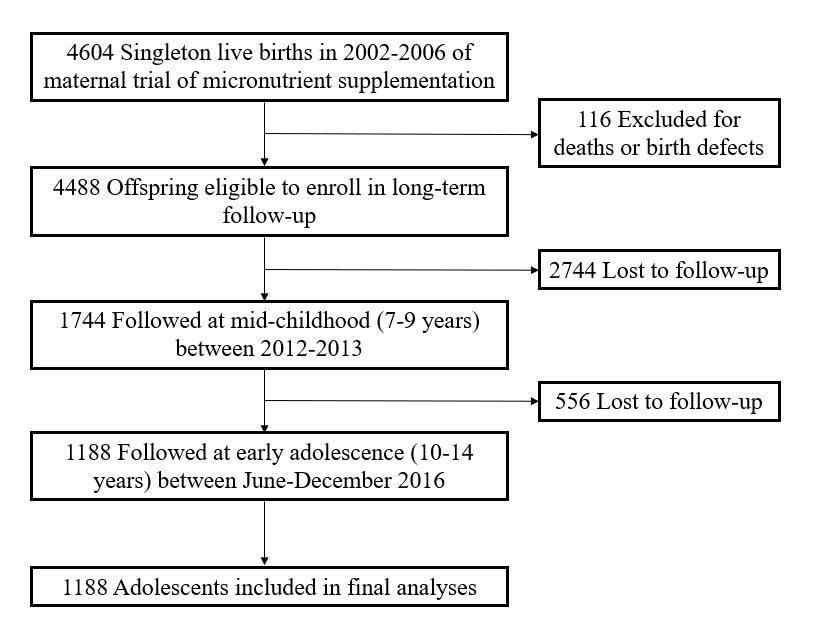


Supplemental Figure 1 Participant flowchart of a birth cohort in rural western China.


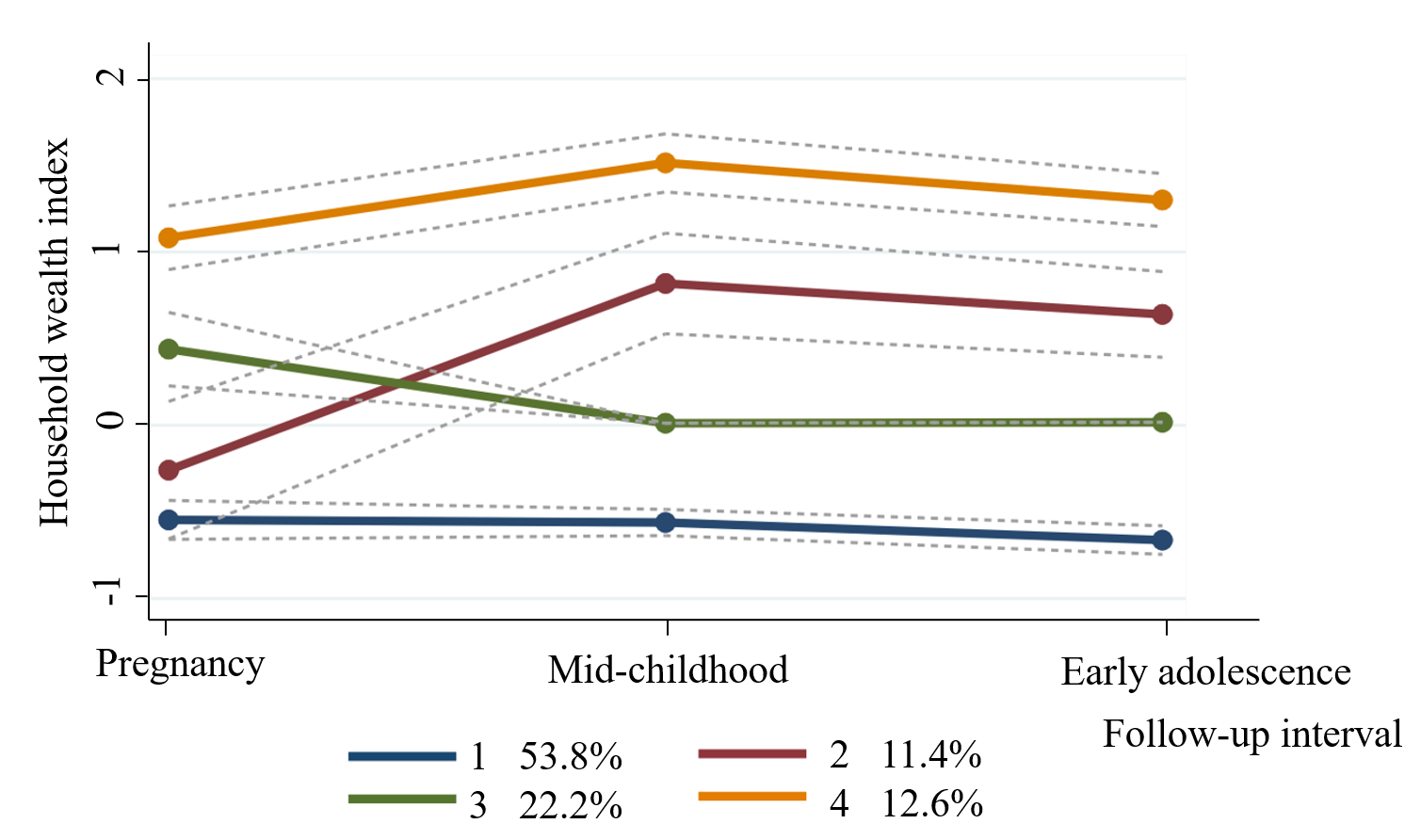


Supplemental Figure 2 Life-course trajectories of household wealth index (relative-scale mobility) from pregnancy to early adolescence in a birth cohort in rural western China (*n*=1188). Lines show for each trajectory the predicated means of *z* score and 95% confidence limits. We identified four distinct trajectories of household wealth from pregnancy to early adolescence. The Subgroup i, ii, iii and iv were labeled “Consistently low”, “Upward”, “Downward” and “Consistently high”, and the corresponding sample size percentage was presented in the bottom of the figure.
